# New Polycaprolactone-Containing Self-Healing Coating Design for Enhance Corrosion Resistance of the Magnesium and Its Alloys

**DOI:** 10.3390/polym15010202

**Published:** 2022-12-31

**Authors:** Andrey S. Gnedenkov, Sergey L. Sinebryukhov, Valeriia S. Filonina, Alexander Yu. Ustinov, Sviatoslav V. Sukhoverkhov, Sergey V. Gnedenkov

**Affiliations:** Institute of Chemistry, Far Eastern Branch of the Russian Academy of Sciences, Vladivostok 690022, Russia

**Keywords:** magnesium, alloy, biodegradation, protective properties, plasma electrolytic oxidation, hybrid coatings, electrochemistry, HBSS, corrosion inhibitor, polycaprolactone, additive technology

## Abstract

The method of hybrid coating formation on the surface of a bioresorbable wrought magnesium alloy and magnesium obtained by additive technology was proposed. Plasma electrolytic oxidation (PEO) with subsequent treatment of the material using an organic biocompatible corrosion inhibitor and a bioresorbable polymer material was used to obtain the protective layers. The optimal method of surface treatment was suggested. Using SEM/EDX analysis, XRD, XPS, and confocal Raman microspectroscopy, the composition of the formed surface layers was determined. The corrosion protection performance of the formed coatings was studied by potentiodynamic polarization and electrochemical impedance spectroscopy techniques in 0.9 wt.% NaCl and HBSS. Hydrogen evolution and mass loss tests were performed to study the corrosion rate of samples with different types of protective coatings. Sealing the pores of PEO coating with a polymeric material contributes to a significant reduction in the amount of the inhibitor diffusing into a corrosive medium. The best barrier properties were established for the hybrid coating formed with a one-stage application of benzotriazole and polycaprolactone. Such layers reduce the rate of alloy degradation due to active protection.

## 1. Introduction

Metallic biodegradable implants for osteosynthesis should provide not only sufficient mechanical strength throughout the healing period but also good adhesion and proliferation of cells. The combination of these features can be achieved by both the selection of a material with mechanical characteristics close to the ones of human bone and surface treatment that can preserve the implant mechanical integrity during the bone tissue healing period and ensure complete resorption of its degradation products [1]. Among other biocompatible metals, magnesium and its alloys are the most promising due to the combination of mechanical characteristics (high specific strength and Young’s modulus close to bone tissue), as well as the ability to spontaneously degrade in saline solutions (in particular, in the human body). However, the rate of magnesium resorption is too high to ensure the mechanical integrity of the implant throughout the process of osteosynthesis [2,3] For this reason, it is necessary to find an effective and affordable way to control the materials’ degradation rate, ensuring a uniform course of corrosion processes over the implant surface during the healing period and moderate hydrogen release. One of the methods for reducing the electrochemical activity of magnesium as an implantation material is the application of protective (including biodegradable) coatings. Among other methods for forming coatings on the surface of magnesium and its alloys (electrochemical [4] and electrophoretic [5] deposition, sol-gel method, sputtering, and laser deposition [4]), the most accessible and technologically advanced method is plasma electrolytic oxidation (PEO) [6,7,8,9,10]. This method allows the formation of multifunctional heterostructural layers with specified (by varying the electrolyte composition and oxidation mode parameters) properties on the surface of valve metals (in particular, magnesium and its alloys). Nevertheless, in spite of an increase in the corrosion resistance of the Mg alloy sample after PEO treatment, the protective properties of the coated specimen may not be enough for its reliable application as the implant material. The reason for the rapid deterioration of the protective properties of the PEO layer is its highly porous structure, which provides fast penetration of the aggressive medium to the substrate material. Pores and microdefects of an oxide layer can become centers of premature initiation and the development of corrosion processes on the magnesium surface. This results in pitting formation, and finally, the loss of mechanical integrity is realized. Therefore, the structure of the base PEO coating should be modified to prolong the period of material exploitation [6,11,12,13].

At the same time, the features of a coating’s surface relief can be used as positive factors. In particular, deep pores in the outer part of the PEO coating can act as nano- and microcontainers for further modification of the PEO layer with various protective agents, providing the resistance of a material to electrochemical reactions that cause its premature degradation [14,15]. The goal of improving the protective properties of coatings on resorbable implants is slowing down the corrosion rate. Therefore, nontoxic biocompatible corrosion inhibitors, which impart a self-healing property to the formed layers, are the most suitable for improving the barrier properties of the magnesium implants. According to the analysis of literature data, the application of inhibitor-containing protective coatings is a common method of corrosion protection due to its effectiveness and ease of use [16]. For example, benzotriazole is a low-toxicity antibacterial agent (median lethal dose LD_50_ = 965 mg/kg [17]) and is widely used as a corrosion inhibitor for various nonferrous metals. Lamaka et al. [18] studied the efficiency of different corrosion inhibitors of magnesium and showed that benzotriazole at a low concentration (0.005 M) can decrease the corrosion rate of the material by blocking the cathodic activity of Cu, Ni, and Fe in the composition of the alloy due to the chelating mechanism. The study [19] showed that benzotriazole can increase the corrosion resistance of an Mg alloy by promoting the nucleation sites to initiate the formation of crystalline Mg(OH)_2_. It was established [20] that benzotriazole at the concentration of 0.02 M is an effective corrosion inhibitor that provides uniform magnesium dissolution during the corrosion process. The work [21] presents a method for protecting the surface of the AZ31B magnesium alloy by forming an anode film in electrolytes containing benzotriazole in various concentrations (3 g/L, 5 g/L, and 10 g/L). It is reported that the coating formed in an electrolyte containing 5 g/L of benzotriazole is characterized by the highest surface relief uniformity, the smallest pore size, and the best corrosion resistance. The study [22] dealt with the formation of protective PEO coatings on magnesium alloy AM50. Sun et al. performed oxidation in silicate-fluoride electrolytes containing halloysite nanotubes (HNTs) and HNTs loaded with benzotriazole. As a result of the evaluation of the electrochemical properties of the formed coatings, it was found that the incorporation of benzotriazole into their composition not only contributes to an increase in corrosion resistance but also imparts the self-healing property. Therefore, benzotriazole can be used as an efficient inhibitor agent suitable for corrosion protection of promising biomedicine magnesium alloys.

In the course of the analysis of literature data on the topic of this study, it was revealed that there were no works devoted to the assessment of the complex effect of the action of benzotriazole in combination with a biodegradable oxide coating obtained by the PEO method on increasing the protective properties of magnesium and its alloys promising for implant surgery. The presented study dealt with the development of a new method for reducing the rate of corrosion degradation of magnesium by forming biocompatible PEO coatings modified with benzotriazole. In order to ensure the prolongation of the mechanism of active corrosion protection by controlling the release of an inhibitor from the pores of an oxide layer, the resulting microcontainers (pores of the PEO coating) were sealed by treating with a bioresorbable polymer material—polycaprolactone (PCL). It is stated [23] that PCL is a biodegradable polyester that has been actively used in biomedical applications since the 1970s. PCL is a nontoxic polymer, which produces carbon dioxide (CO_2_) and water (H_2_O)—products harmless to human body degradation [24]. PCL is applied in many studies [25,26,27,28,29] and clinical practice, and according to the Food and Drug Administration (FDA), it is a bioresorbable polymer [30]. This polymer is applied in the production of tissue scaffolds [31]. The application of polycaprolactone in the biomedical field is also shown in [32,33]. Moreover, since PCL undergoes degradation in corrosive media, it can be used for the delivery of drugs or other chemical substances [23]. Therefore, the combination of PCL and a corrosion inhibitor can promote a sufficient increase in the protective properties of biodegradable Mg implants.

## 2. Materials and Methods

### 2.1. Samples and Processing Procedures

#### 2.1.1. Sample Preparation

The studies were carried out on metal plates 15 × 20 × 1.5 mm in size, made of wrought magnesium alloy MA8 (wt.%: Mn—1.3–2.2, Ce—0.15–0.35, Zn—0.3, Si—0.1, Al—0.1, Fe—0.05, Cu—0.05, Ni—0.007, Be—0.002, Mg—balance). Moreover, plates obtained by direct laser deposition (DLD) of MPF-4 magnesium powder (Russian GOST 6001-79) [34] (AT-Mg) were used.

AT-Mg samples were obtained at the Center of Laser Technologies (Institute of Automation and Control Processes of FEB RAS) on a setup that included an LS-1-K fiber laser complete with an IPG FLW-D50V welding head and industrial robot KUKA KR-30 HA with a rotary inclined positioner KUKA DKP-400 and a system for supplying process gases to the deposition zone. The formation of the samples was carried out in a protective sealed stainless steel chamber with a constant supply of argon (Ar—99.993%; Russian GOST 10157-79), which ensured a stable process of direct laser deposition. This chamber allows working with magnesium and provides a controlled protective gas atmosphere. When forming AT-Mg samples, a gas–powder mixture of MPF-4 powder and helium was used to supply the material to the place of deposition. The gas–powder mixture was supplied into the melt bath from two nozzles of the feed module. A protective process gas (argon) was supplied through two other nozzles of the module. The samples were formed in 30 passes using a laser beam with a diameter of 2.5 mm at a speed of 5 mm/s in 0.5 mm steps and a laser power of 240 W. As a result of DLD, magnesium samples of 20 × 20 × 0.7 mm in size were obtained. The detailed description of the DLD process can be found elsewhere [34].

The samples were ground on a TwinPrep 5× grinding/polishing machine (Allied High Tech Products, Inc., Compton, CA, USA) using silicon carbide (SiC) grinding paper with a gradual decrease in the abrasive grain size to 14–20 μm (P1000). Then, the specimens were washed with deionized water and dried in a desiccator at 40 °C.

#### 2.1.2. PEO Coating Formation

Formation of the base hetero-oxide layer was carried out using a plasma electrolytic oxidation unit with automated program control. Oxidation was carried out in a two-component electrolyte containing 20 g/L Na_2_SiO_3_ and 5 g/L NaF. The samples were polarized in a bipolar mode in two stages. In the first stage, the anode component increased potentiodynamically from 0 to 280 V for 200 s with a sweep rate of 1.4 V/s. The cathode component was maintained potentiostatically at −40 V. In the second stage, the anode and cathode components were changed potentiodynamically to a voltage of 200 V and −10 V, respectively, for 400 s. The sweep rate was equal to 0.2 V/s and 0.075 V/s, respectively. The duty cycle was equal to 1.

#### 2.1.3. Inhibitor and Polymer Treatment

For the presented study, various coatings contained either a corrosion inhibitor or a polymer on the surface of the PEO layer (composite coatings), and there were ones including both an inhibitor and a polymer (hybrid coatings). The PEO layer was impregnated with a corrosion inhibitor from an aqueous solution of benzotriazole (Btr) prepared by dissolving it in deionized water at concentrations of 0.05 M and 0.1 M with constant stirring at room temperature. Further, the pH of all solutions was neutralized (pH = 7.0–7.2) by alkalization with sodium hydroxide (NaOH). PEO-coated samples were treated by immersing in the solution under vacuum using an Epovac vacuum impregnation apparatus (Struers, Denmark) in order to ensure the best filling of the pores of the PEO layer with the inhibitor. Further processing included dipping in solutions for 1 h to increase the amount of the inhibitor in the outer porous part of the PEO layer, as well as to ensure the adsorption of Btr on the bottom of the pores in the oxide coating.

A concentration of 0.05 M of an inhibitor is the standard used in many studies by various scientific groups [18,35,36,37]. Moreover, in one work [18], a comprehensive screening of various Mg corrosion inhibitors was presented, where in most cases, a 0.05 M solution of inhibitor was used. Therefore, to have the possibility to make a proper and fair comparison of inhibitor efficiency, it is suggested to use one unified concentration (0.05 M).

The concentration of 0.1 M was used in this work for comparison of the effect of the inhibitor’s quantity in the solution for impregnation on the corrosion resistance of the coated Mg alloy.

To seal the microcontainers, a solution of polycaprolactone (PCL) (60 g/L) in dichloromethane was prepared. The formation of the surface layer was carried out by dipping inhibitor-loaded samples into the polymer solution, holding for 5–7 s, and slowly withdrawing. The polymer was applied twice. Between repeated applications, the samples were dried in an oven (*t* = 40 °C for 24 h).

**Figure 1:**
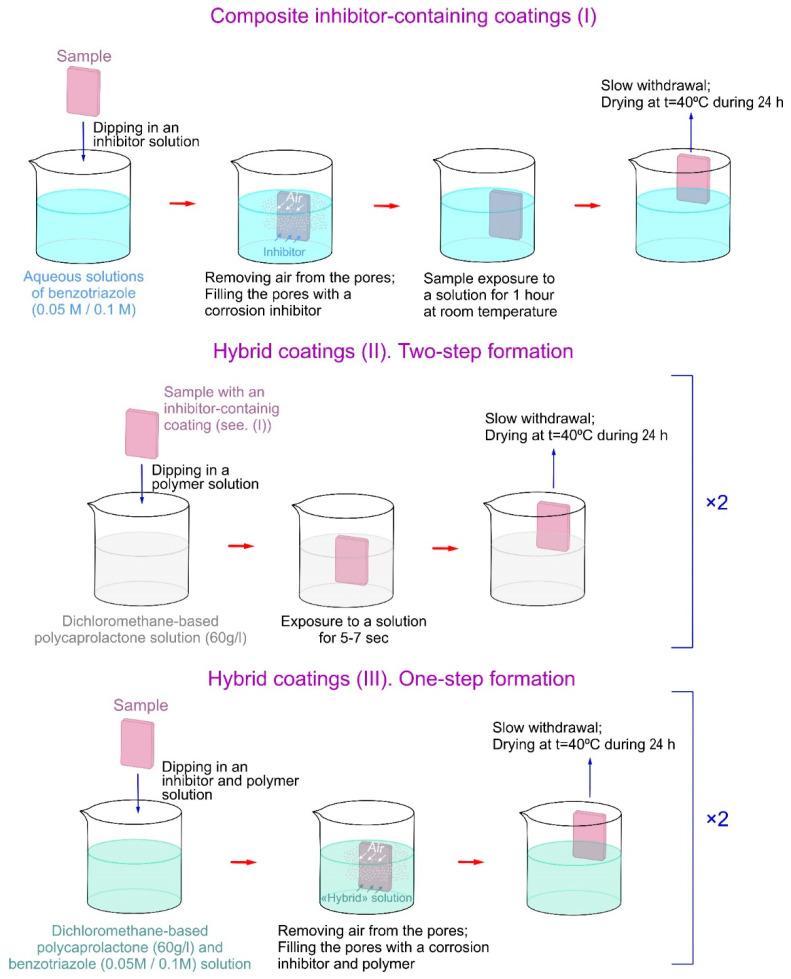
The scheme of the formation processes for various types of the studied coatings.

As an alternative to the stepwise application of inhibitor-containing coatings, hybrid coatings were formed by treating the PEO layer with 0.05 M or 0.1 M benzotriazole solutions in dichloromethane containing 60 g/L of polycaprolactone. Modification with the resulting solution was carried out using a vacuum impregnation apparatus in order to extract air from the pores and for the most complete incorporation of the inhibitor into the heterostructure of the surface layer. All the samples were dried in a desiccator at *t* = 40 °C for 24 h. The process of formation of the composite and hybrid layers is shown in Figure 1.

The types of samples prepared for the research are described in Table 1.

The photographs of the main test samples (without coating, with PEO layer, and with CC-0.05 and HC-0.05-2 coatings) are shown in Figure S1.

#### 2.1.4. Cross-Section Preparation

Samples for cross-section preparation were embedded in epoxy resin (EpoxySet #145-20005, Allied High Tech Products Inc., USA). The diameter of a FixiForm casting mold (Struers A/S, Denmark) was 30 mm. The next step was mechanical processing on a Tegramin-25 grinding and polishing machine (Struers A/S, Denmark). Grinding and polishing stages included processing using paper for fine grinding (SiC Foil, MD-Largo) and polishing discs (MD-Mol, MD-Chem), as well as diamond suspensions (DP-Suspension P) with an abrasive size of 9 µm and 3 µm together with DP-Lubricant Brown. The final step was polishing with OP-S NonDry colloidal suspension.

### 2.2. Surface Characterization

#### 2.2.1. SEM-EDX

A study of the morphology of the obtained coatings and the distribution of elements within the thickness of the samples was performed by scanning electron microscopy (SEM) and energy dispersive analysis (EDS) using a Merlin Gemini 2 device (Carl Zeiss Group, Jena, Germany) with a Silicon Drift Detector X-MaxN 80 (Oxford Instruments NanoAnalysis, Concord, MA, USA).

#### 2.2.2. XRD Analysis

X-ray diffraction analysis (XRD) of PEO-coated samples was carried out using a SmartLab diffractometer (Rigaku, Japan) with CuK_β_ radiation (Bragg–Brentano geometry). The spectrum was recorded in the range 2θ = 4–90° with a step of 0.01° at room temperature. A generator current of 140 mA and a voltage of 42 kV were used.

#### 2.2.3. XPS Analysis

X-ray photoelectron spectroscopy (XPS) was used for chemical analysis of the composite coating formed on the surface of the magnesium alloy MA8 with a PEO layer impregnated with a 0.1 M solution of benzotriazole. A spectrometric complex SPECS (Germany) with a hemispherical electrostatic energy analyzer PHOIBOS-150 was used to study the surface under ultrahigh vacuum (0.5 µPa). Nonmonochromatic AlKα radiation with an energy of 1486.6 eV was used. All spectra were calibrated against the aliphatic carbon line (C1s line) with a binding energy of 285.0 eV. To determine the chemical composition of the deeper layers of the protective coating and to clean the surface, the sample was etched with an Ar^+^ for 10 min at an energy of 5000 eV. As a result of ion etching, a layer with a thickness of about 5–10 nm was removed.

#### 2.2.4. Raman Spectroscopy

Confocal Raman microspectroscopy was used to confirm the impregnation of a PEO coating with a corrosion inhibitor. The inhibitor distribution over the surface layer was estimated for CC-0.1 Mg alloy samples. Micro-Raman scattering spectra were obtained in the range from 300 to 1450 cm^−1^ using an Alpha 500 confocal spectrometer (WITec, Germany) and WITec Control software. The Raman spectrum was recorded using a green laser (wavelength 532 nm) with a radiation power of 30 mW for 10 min (60 accumulated spectra). The distribution of benzotriazole over the surface of the PEO layer was presented as 2D maps. The maps were designed as a result of scanning a selected coating area with dimensions of 35 × 30 μm containing 35 × 30 spectra. The integration time for spectrum recording during the scanning mode was 1 s.

### 2.3. Corrosion Studies

#### 2.3.1. Electrochemical Measurements

A preliminary assessment of the protective properties of the obtained surface layers was performed using traditional electrochemical methods—potentiodynamic polarization (PDP), electrochemical impedance spectroscopy (EIS), as well as monitoring the open circuit potential (OCP) of the sample during exposure to the solution. The experiments were carried out using the VersaSTAT MC electrochemical system (Princeton Applied Research, San Jose, CA, USA). The electrolyte was 0.9 wt.% NaCl aqueous solution. The surface area of the studied sample was equal to 1 cm^2^. A platinized niobium mesh served as a counter electrode, and a silver chloride (Ag/AgCl) electrode was the reference one (the potential versus standard hydrogen electrode is equal to 0.197 V). Electrochemical tests (EIS and PDP) were carried out after preliminary exposure of the specimen to an electrolyte for 60 min. The duration of EIS measurements in NaCl solution was 24 h (spectrum was recorded every 2 h). PDP measurements were also carried out after the last impedance spectrum was taken (after 24 h of exposure). The sweep rate during the PDP test was equal to 1 mV/s. The sample was polarized in the anodic direction in the potential range from *E_C_* − 0.25 V to *E_C_* + 0.5 V. The frequency during the recording of the impedance spectra varied in the range from 1 MHz to 0.1 Hz with a logarithmic sweep of 10 points per decade.

Long-term electrochemical tests were carried out in Hanks’s balanced salt solution (HBSS). Tests in HBSS (Gibco, UK, # 14025) were performed for 7 days. The impedance spectra were recorded every 2 h for the first 2 days and then every 4 h. To determine the values of corrosion potential *E_C_* and corrosion current density *I_C_*, the Levenberg–Marquardt approach was used as the most suitable one for describing the electrochemical parameters of metals with an oxide layer on the surface (in particular, magnesium and its alloys) [21,38,39]. Modeling of charge transfer processes at the electrode/electrolyte interface was performed during an analysis of EIS spectra by the fitting of the experimental spectrum using the appropriate equivalent electrical circuits (EECs). The errors for the parameters calculated using EEC were <5%. The chi-square value was about χ^2^ = 1 × 10^−4^.

#### 2.3.2. Gravimetric and Volumetric Analysis

To establish the corrosion rate of samples with protective coatings, gravimetric and volumetric methods were used. These methods are based on determining the material mass loss and the volume of the released hydrogen per unit area of the sample during exposure to Hanks’ solution for 7 days. The material’s mass loss as a result of corrosion was determined by the gravimetric method after the removal of corrosion products by washing the samples for 15 min in deionized water using an ultrasonic bath. After washing, the samples were weighed on an AUW120D analytical balance (Shimadzu, Kyoto, Japan). For volumetric tests, a eudiometer (art. no. 2591-10-500 from Neubert-Glas, Germany), excluding the contact of the test solution with the air atmosphere, was used.

Four samples were used simultaneously for exposure to a corrosive environment. The size of each sample was 15 × 20 × 1.5 mm, and the total surface area was 28 cm^2^ (the area of each sample was 7 cm^2^). The experiment was carried out at room temperature [40]. The volume of the testing solution was 300 mL. All experiments were carried out three times to assess the reliability of information. The solution was stirred at a constant speed (350 ± 100) rpm. At the end of the test, the samples were removed from the solution, washed with deionized water, and dried in air.

#### 2.3.3. Inhibitor Release Test and Wettability Analysis

In order to establish an inhibitor release process from the composition of the formed inhibitor-containing protective layers, the high-performance liquid chromatography (HPLC) technique was used. Samples with composite (CC-0.1) and hybrid coatings (HC-0.1-2) were studied. Five specimens of each type (the size of one sample was 15 × 20 × 1.5 mm) were immersed in 300 mL of 0.9 wt.% NaCl solution for 21 days. An amount of 3 mL was sampled every 24 h to assess the concentration of the inhibitor released into solution during the exposure of specimens. HPLC analysis was performed on a Shimadzu LC-20A liquid chromatograph with an SPD-20A UV detector (detector wavelengths were 200 and 210 nm) and a low-temperature laser light scattering detector ELSD-LT II.

Compound separation was carried out on an Agilent Eclipse XDB-C18 column (4.6 × 150 mm, 5 µm). The column temperature was equal to 40 °C. Methanol/0.03 M acetic acid (pH 2.9) 95:5 was used as the eluent. The flow rate was 1 mL/min.

The evolution of the surface wettability after PEO layer and hybrid coating formation was studied using the sessile drop method using a DSA100 device (Krüss, Germany) at room temperature. The volume of the distilled water drop was 5 µL.

## 3. Results and Discussion

### 3.1. Coatings Obtained on MA8 Magnesium Alloy

#### 3.1.1. Structure and Composition

**Figure 2:**
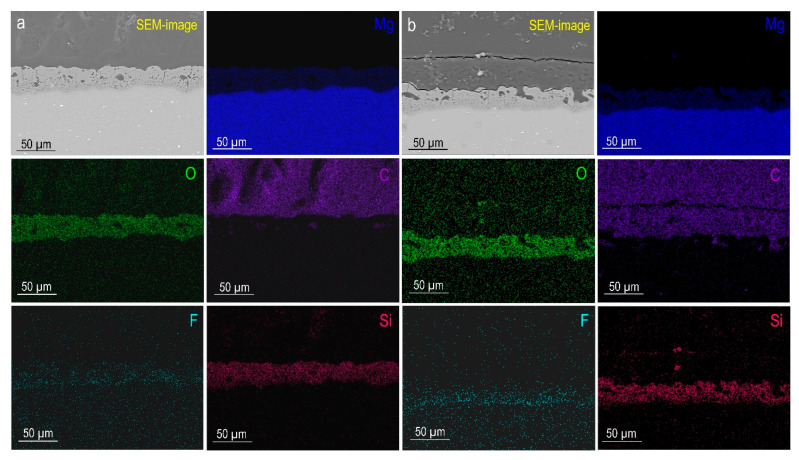
SEM images of cross-sections and EDX maps of elements’ distribution within the thickness of MA8 alloy sample with PEO coating (**a**) and CC-P coating (**b**).

**Figure 3:**
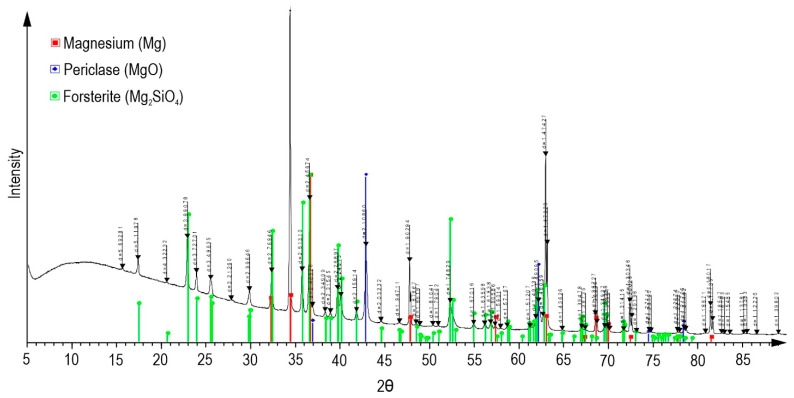
X-ray diffraction pattern of the sample with PEO coating.

As a result of plasma electrolytic oxidation, a ceramic-like coating was formed on the MA8 magnesium alloy. The morphology of the obtained surface layers is characterized by the presence of numerous pores and microdefects (Figure 2a). Based on the data of energy dispersive X-ray analysis (Figure 2a), it can be concluded that the composition of the base PEO layer is characterized by the presence of magnesium, oxygen, fluorine, and silicon, which are native components of the human body. According to the data obtained by the XRD method, the composition of oxide coatings includes magnesium oxide (periclase, MgO) and magnesium orthosilicate (forsterite, Mg_2_SiO_4_) (Figure 3). The presence of magnesium (Mg) peaks on the diffraction pattern is due to the penetration of X-rays through the PEO layer, since the coating possesses low reflective ability and a highly porous structure [22,41]. This combination provides XRD analysis to detect the material substrate. It is a typical effect for PEO coatings [42,43,44]. It should be noted that metallic Mg cannot be in the content of the protective layer, since the PEO process is realized at a very sharp condition in the electrolyte (the temperature and pressure in the spark plasma channel can reach 10,000 K and 100 MPa, respectively). Therefore, such electrochemically active metal as magnesium is immediately oxidized and presented in the coating as magnesium oxide. Therefore, EDX data show the presence of Mg in the form of oxide and silicate, which is in agreement with XRD data.

Figure 2b and Figure S2–S4 present SEM images and EDX maps of elements’ distribution within the thickness of the formed coatings. The presence of corrosion inhibitors in the composite coatings was not detected by SEM/EDX. The analysis of the results indicates a high pore occupancy of the PEO coating with a polymer component and solutions containing both an inhibitor and polymer. The presence of these modifying agents is observed on the surface and in the pores of the PEO layer. As a result of this treatment, the probability of penetration of an aggressive medium down to the magnesium substrate is significantly reduced, and, accordingly, the level of corrosion resistance of the material increases.

**Figure 4:**
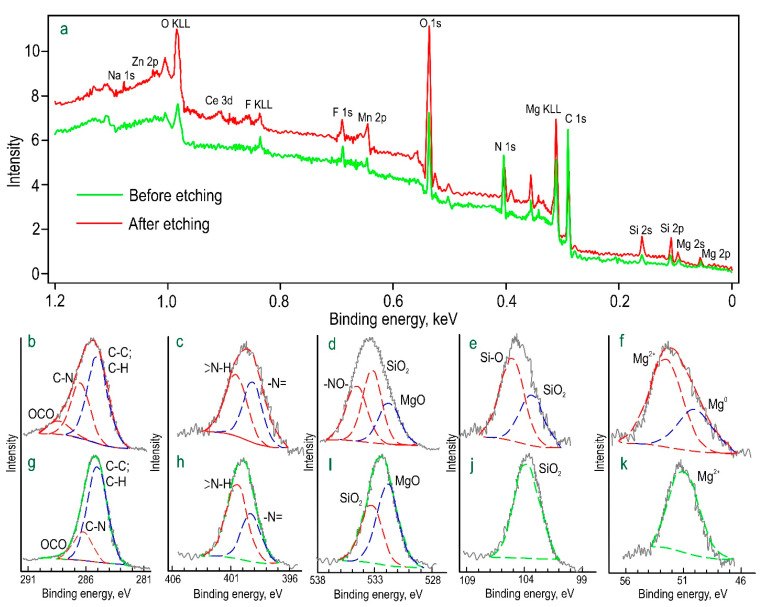
XPS spectra obtained for a sample with CC-0.1 coating (**a**). The detailed high-resolution spectra of C 1s (**b**,**g**), N 1s (**c**,**h**), O 1s (**d**,**i**), Si 2p (**e**,**j**), and Mg 2p (**f**,**k**) before (**g**–**k**) and after (**b**–**f**) Ar^+^ etching.

X-ray photoelectron spectroscopy (XPS) was used to determine the chemical composition of the formed protective layers, taking into account the presence of a corrosion inhibitor. Samples with CC-0.1 composite coating were studied by the XPS method (Figure 4; Table 2). Analysis of the surface layer of the sample with CC-0.1 before etching indicates the presence of a large amount of aliphatic carbon (C–C, C–H, *E_b_* = 285.0 eV) due to probable contamination of the surface (as follows from the composition of the layer after etching). The binding energies of 400 and 399 eV are related to nitrogen of both “pyrrole” and “pyridine” types, which is due to the presence of these heteroatoms in the benzotriazole compound. A significant amount of carbon with *E_b_* = 286 eV is explained by the C–N bond, which is characteristic of the benzotriazole structure.

The presence of alloying (Mn and Ce) included in the PEO layer was established in the coating composition. The presence of sodium, fluorine, oxygen, and silicon is related to the composition of the PEO electrolyte and the possible formation of sodium silicate and magnesium fluoride in the protective layer. These compounds (unlike magnesium silicate and magnesium oxide) could not be detected by X-ray diffraction due to their low concentration or presence in the coating composition in an X-ray amorphous state.

After etching the top layer, a significant increase in the content of oxygen, silicon, and magnesium is observed in the composition of the coating, and the content of aliphatic carbon significantly decreases. The obtained result indicates the presence of magnesium silicate, which is a part of the base PEO coating. The analysis of experimental data allows us to conclude that the content of the main elements (C and N) and their chemical state in the upper layer of CC-0.1 are consistent with the stoichiometry of benzotriazole.

**Figure 5:**
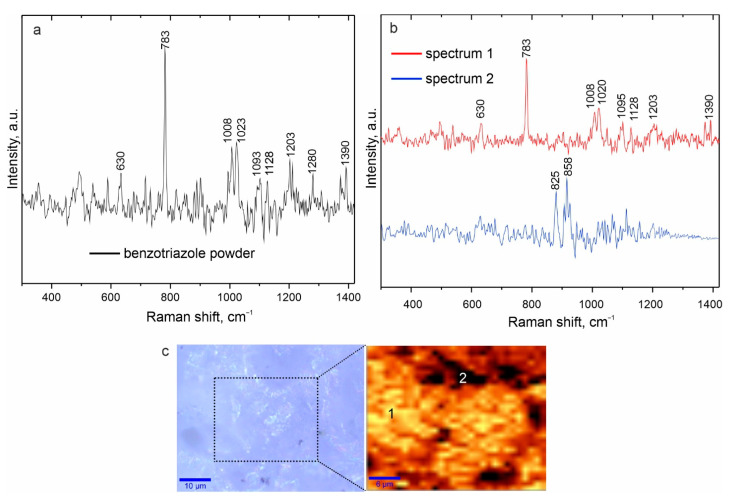
Raman spectrum of benzotriazole powder (**a**), Raman spectra collected in the areas (marked with numbers 1 and 2 in (**c**)) of the sample coated with CC-0.1 (**b**), optical image and 2D inhibitor distribution map (**c**).

The chemical composition of the CC-0.1 composite coating was also studied using confocal Raman microspectroscopy. This method in the scanning mode additionally allows the determination of the distribution of one of the protective layer components at the microlevel. Figure 5a shows the Raman spectrum of benzotriazole. Analysis of this spectrum indicates the bands corresponding to the corrosion inhibitor. The intense characteristic peak responsible for the so-called “breathing” vibrations of the C=C bonds of the benzene ring was recorded at 783 cm^−1^. Peaks at 1008 cm^−1^ and 1023 cm^−1^ are associated with bending vibrations of bonds (caused by stretching the skeletal bonds C=C in the benzene ring, as well as δ(CH)) [45,46]. Peaks at 1093 cm^−1^ and 1128 cm^−1^ refer to bending vibrations δ(NH) and δ(CH), respectively. The narrow peak at 1203 cm^−1^ is responsible for the combination of asymmetric stretching vibrations v(N–N–N) and bending vibrations δ(NH). The band at 1280 cm^−1^ characterizes the stretching vibrations of skeletal bonds, bending vibrations δ(NH), and δ(CH). Peaks at 630 cm^−1^ and 1390 cm^−1^ are attributed to the torsional and stretching vibrations of bonds in the triazole ring [45,46,47].

To determine the distribution of benzotriazole over the surface of the CC-0.1 sample, a 2D map was designed using a filter in the spectral range of 750–850 cm^−1^ corresponding to the characteristic peak for Btr (“breathing” vibrations of C=C bonds of the benzene ring at 783 cm^−1^). Figure 5c depicts the optical image of the coating’s studied area (highlighted by a frame) and a 2D map of the benzotriazole distribution over the surface of the PEO layer. An analysis of the experimental data indicates a nonuniform distribution of the inhibitor over the coating surface. The area of light zones (zones with a high concentration of benzotriazole) is significantly higher than the dark ones. The lower concentration of the inhibitor recorded in the dark areas may be due to the complex coating’s morphology and deposition of most of the inhibitor in the pores of the outer layer. Numbers 1 and 2 mark the surface areas corresponding to regions with high and low inhibitor content, where the spectra shown in Figure 5b are recorded (spectra 1 and 2).

The bands on spectrum 1 are clearly distinguishable at 630, 783, 1008, 1020, 1095, 1128, 1203, and 1390 cm^−1^ corresponding to bond vibrations in benzotriazole. At the same time, spectrum 2 shows other peaks at 825 and 858 cm^−1^, responsible for the bonds in tetrahedral structures of silicates [48,49], due to the presence of magnesium silicate in the composition of the PEO layer, established by the XRD method.

#### 3.1.2. Electrochemical Properties

**Figure 6:**
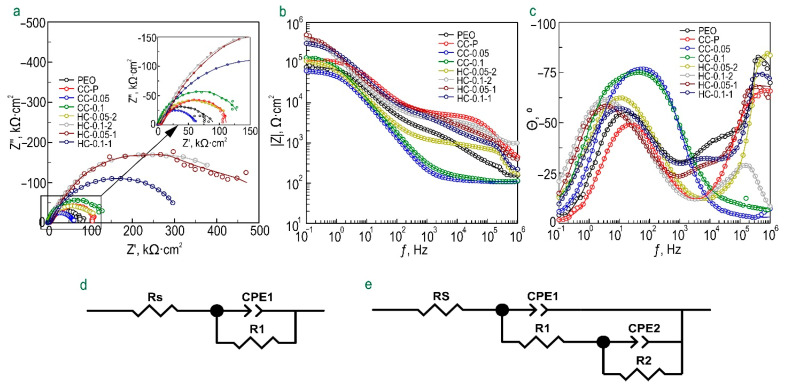
Impedance spectra presented in the form of Nyquist (**a**) and Bode (**b**), (**c**) diagrams after 1 h exposure of samples with benzotriazole-containing coatings to 0.9% NaCl solution. Equivalent electrical circuits (EECs) used to fit the experimental impedance spectra (**d**,**e**).

**Figure 7:**
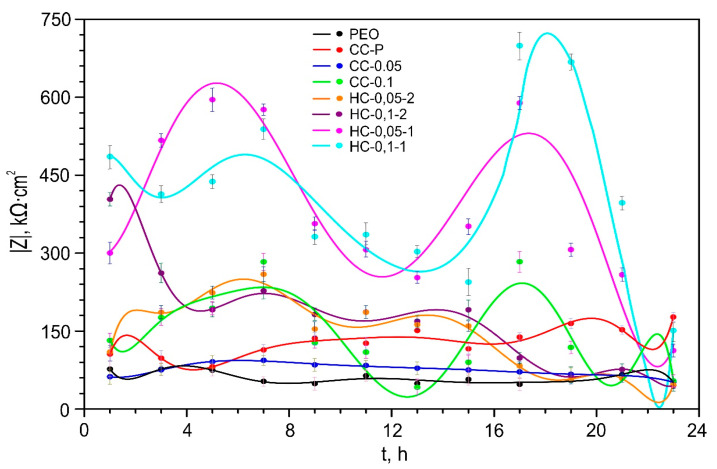
Dynamics of change in magnitude of impedance modulus, measured at a low frequency (|Z|*_f_*_=0.1Hz_), over the exposure time of samples with different types of Btr-containing coatings in sodium chloride solution. The errors for the measured parameters did not exceed 10%.

The corrosion behavior of the studied samples with formed protective layers after their exposure to 0.9 wt.% NaCl solution for 1 h is shown on the impedance spectra presented in Nyquist and Bode plots (Figure 6). The evolution of the impedance modulus measured at the lowest frequency during the sample exposure to the studied medium is presented in Figure 7.

Bode plots representing the dependence of the phase angle (*θ*) on frequency (ƒ) for the PEO coating, CC-P, and all types of hybrid benzotriazole-containing coatings are characterized by the presence of two time constants (Figure 6c), which determines the fitting of the impedance spectra using an equivalent electrical circuit (*EEC*) with two series–parallel-connected *R-CPE* chains (Figure 6e, Tables S1 and S2). The use of the constant phase element (*CPE* element) instead of the ideal capacitance (*C*) is due to the inhomogeneity of the coatings under study. *R*_1_–*CPE*_1_ characterizes the resistive component (*R*_1_) of the outer (porous) layer of the blank PEO coating or PEO coating treated with a polymer component (CC-P) or with an inhibitor–polymer solution (HC-0.05-1 and HC-0.1-1), as well as the geometric capacitance (*CPE*_1_) of the coatings. The *R*_2_ and *CPE*_2_ elements describe the resistive and capacitive components of an inner (nonporous) sublayer of the coating, taking into account the inhibitor and polymer deposited at the bottom of the pores.

In turn, the *θ-ƒ* plots for composite inhibitor-containing coatings (CC-0.05, CC-0.1) are characterized by the presence of one time constant. This indicates the formation of a uniform surface layer by filling the pores of the base PEO coating with a corrosion inhibitor. For this reason, these impedance spectra were fitted using *EEC* with one parallel *R-CPE* chain (Figure 6d). It should be noted that fitting the impedance spectra using an *EEC* with two *R-CPE* chains led to a decrease in the chi-squared parameter. This configuration of *EEC* elements (*R-CPE*) characterizes the resistance (*R*) of the benzotriazole-impregnated coating and the capacitance behavior (*CPE*) of an entire surface layer.

Samples with all types of benzotriazole-containing coatings are also characterized by an insignificant change in the values of the *CPE* coefficient (*Q*) and the exponential factor (*n*) during the whole sample’s exposure time. This indicates a slight change in the morphology, composition, and properties of the studied surface layers. For hybrid coatings, a significant increase in the resistance of the outer (*R*_1_) and inner (*R*_2_) layers of a protective coating was revealed as a result of the modification of the pores of the base PEO layer with a corrosion inhibitor and polymer. The evolution in these parameters during the exposure time of the sample (alternate increase and decrease) to a sodium chloride solution indicates the realization of the self-healing effect of the formed coatings. The initial total resistance of the protective PEO coating (*R*_1_ + *R*_2_, 80 kΩ·cm^2^) increased up to 47% and 78% after polymer (118 kΩ·cm^2^) and inhibitor (142 kΩ·cm^2^) treatment. Formation of the hybrid coating provides an increase in *R*_1_ + *R*_2_ in the region from 51 to 642% (121–594 kΩ·cm^2^).

As a result of a comparative assessment of the level of protective properties of the studied coatings, a positive effect of benzotriazole’s loading into the composition of the formed layers on their resistance to corrosion processes was established. The value of *|Z|_f_*_=0.1Hz_ for HC-0.1-2 coatings (*|Z|_f_*_=0.1Hz_ = 403,650 Ω·cm^2^) is characterized by more than a three-fold increase in comparison with the value of this parameter for CC-P (*|Z|_f_*_=0.1Hz_ = 105,880 Ω·cm^2^). It should be noted that, for HC-0.05-2, no intensive increase in corrosion resistance was registered, which is probably due to an insufficient concentration of the inhibitor in the composition of the formed coatings. The most obvious manifestation of self-healing properties, and, accordingly, the best corrosion resistance is revealed for samples obtained by one-stage treatment with a dichloromethane-based solution containing a polymer and an inhibitor (HC-0.05-1 and HC-0.1-1) (Figure 6 and Figure 7). The highest value of *|Z|_f_*_=0.1Hz_ was shown by the sample with HC-0.1-1 and reached 699,190 Ω·cm^2^ (17 h of exposure to NaCl solution), which is five times higher than the value *|Z|_f_*_=0.1Hz_ measured at a similar immersion time for the CC-P sample (*|Z|_f_*_=0.1Hz_ = 138,390 Ω·cm^2^).

A comparative evaluation of the PDP test results made it possible to confirm the effectiveness of the proposed method for the formation of inhibitor-containing surface layers (Table 3). The general trend of changes in the level of protective properties of HC samples after 24 h of exposure to a 0.9% NaCl solution is characterized by a slight increase, and in some cases, a significant decrease in the value of the corrosion current density *I_C_*. The best anticorrosion protection was revealed for HC-0.1-1 coatings (*I_C_* = 3.02 × 10^−8^ A·cm^−2^, *R_p_* = 1.84 × 10^6^ Ω·cm^2^ (after 24 h exposure)).

**Figure 8:**
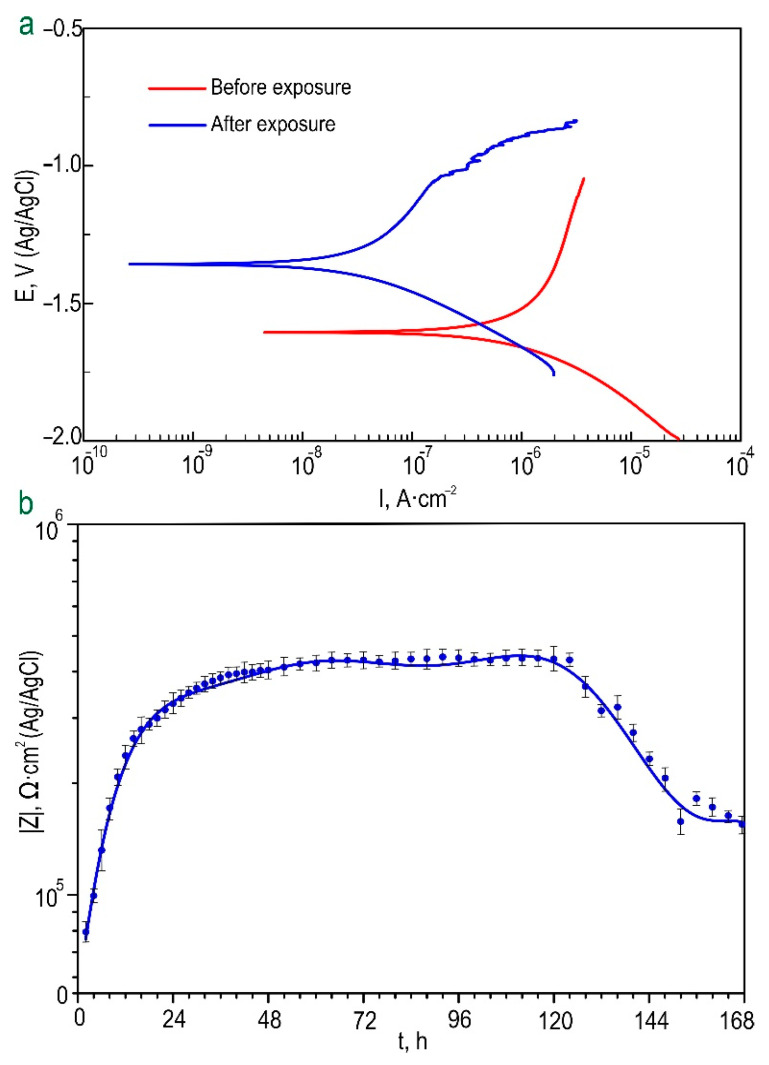
Polarization curves obtained for samples with HC-0.1-2 before and after 7 days of exposure to Hanks’ solution (**a**); dynamics of change in the impedance modulus, measured at a low frequency (*|Z|_f_*_=0.1Hz_) during the exposure time of samples with HC-0.1-2 to Hanks’ solution (**b**).

The results of the preliminary assessment of the electrochemical activity level of samples with HC-0.1-2 by the PDP method in Hanks’ solution are shown in Figure 8a and in Table S3. According to the presented data, the hybrid inhibitor- and polymer-containing coating is characterized by increased resistance to corrosion processes in physiological solution. The evolution of the values of *|Z|_f_*_=0.1Hz_ over the samples’ immersion time in Hanks’ solution (Figure 8b) clearly demonstrates the dynamics of changes in the barrier properties of coatings during long-term contact with HBSS. As a result of a comparative analysis of the obtained data, it was revealed that samples with protective coatings are characterized by stable electrochemical behavior in the studied medium. The high corrosion resistance for the hybrid coating is provided during the first 5 days of exposure, according to presented data. The further process of exposing the samples in solution is characterized by a slight decrease in and stabilization of the level of protective properties. The obtained results are consistent with calculated *EEC* parameters obtained by modeling the impedance spectra of samples with different types of coatings during immersion in Hanks’ solution for 7 days (Table S4). The parameters *CPE* and *R* change insignificantly, confirming the high corrosion resistance of samples with a hybrid layer. As a result of the experiment, it can be assumed that the formed HC-0.1-2 coatings can provide a level of corrosion protection necessary and sufficient to ensure the mechanical integrity of the surgical implant during the initial period of bone tissue formation [50]. Evaluation of the results of PDP experiments after samples’ exposure to Hanks’ solution for 7 days confirmed the effectiveness of the introduction of the inhibitor and polymer into the composition of the formed surface layers (Figure 8a, Table S3). The *I_C_* value for the sample with HC-0.1-2 decreases (*I_C_* = 3.12 × 10^−8^ A∙cm^−2^) in comparison with the corresponding values obtained before exposure to HBSS (*I_C_* = 8.90 × 10^−7^). This confirms the self-healing properties of the obtained surface layers. Such hybrid coatings can provide a stable and long-term implementation of the active protection mechanism of the MA8 magnesium alloy from destructive corrosive influence. A benzotriazole-containing surface layer has high barrier properties and reduces the penetration of aggressive media. As a result of the partial degradation of the upper polymer layer, the adsorbed inhibitor reduces the corrosion rate of the material, providing a slower formation of a corrosion product layer. The decreased rate of corrosion product formation contributes to the appearance of a surface film with higher corrosion resistance in comparison with the film formed during intensive corrosion of the material.

#### 3.1.3. Hydrogen Release and Mass Loss Tests

**Figure 9:**
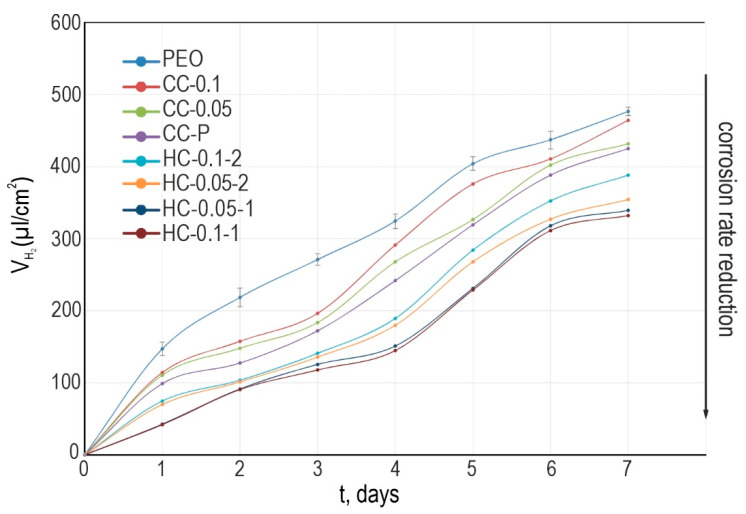
Change in normalized volume of evolved hydrogen ($V_{H_{2}}$) for magnesium alloy MA8 with protective layers during 7 days of exposure to HBSS. The errors for the measured parameters did not exceed 10% (see the error bars for PEO).

An analysis of the experimental results of volumetric measurements of the corrosion rate indicates high protective properties of coatings formed on the basis of the PEO method (Figure 9). The maximum volume of the evolved hydrogen normalized to the sample’s surface area ($V_{H_{2}}$) was recorded for the sample with PEO coating (480 µL/cm^2^). Samples with composite and hybrid protective layers showed a lower corrosion rate. The obtained result indicates a positive effect of surface modification with corrosion inhibitors and polymer materials. Analysis of the obtained data indicates the comparability of results obtained by volumetry and electrochemical impedance spectroscopy (including exposure to 0.9 wt.% NaCl and HBSS). A hybrid coating impregnated with benzotriazole (0.1 M) and polycaprolactone in one stage has the best protective properties. The total volume of $V_{H_{2}}$ for the HC-0.1-1 sample was 310 μL/cm^2^ (1.6 times less than for the base PEO layer). Despite the different dynamics of hydrogen release during the immersion time, the $V_{H_{2}}$ values are close to each other for all samples with protective coatings. This is a consequence of the low degradation rate of the material with anticorrosion coating.

Based on the results of gravimetric tests, the corrosion rate of the samples (mg·cm^−2^·day^−1^) was estimated by calculating their mass loss per surface area, taking into account the exposure time in Hanks’ solution (Table S5). It was revealed that samples with hybrid inhibitor-containing coatings are characterized by increased resistance to corrosion processes during long-term (7 days) exposure to a corrosive environment. Samples with HC-OH 0.1-1 (0.029 ± 0.006 mg·cm^−2^·day^−1^) are characterized by the lowest corrosion rate, which is 1.4 times less than the mass loss of samples with PEO coating (0.041 ± 0.004 mg·cm^−2^·day^−1^). These data are consistent with the results obtained by the potentiodynamic polarization method and electrochemical impedance spectroscopy for the samples exposed to 0.9% sodium chloride solution. It should be noted that for samples with CC-P, HC-0.05-2, and HC-0.1-2, an insignificant weight gain is observed as a result of prolonged exposure to HBSS. This is probably due to the partial degradation of the polymer component in the process of interaction with a corrosive medium and further filling the formed pits with corrosion products. Taking into account the results of EIS and PDP tests, which revealed that the level of samples’ corrosion resistance of HC-0.05-2 and HC-0.1-2 coatings is lower than for samples with HC-0.05-1 and HC-0.1-1, it can be assumed that the duration and efficiency of self-healing of hybrid benzotriazole-containing coatings obtained by two-stage deposition are not enough to provide long-term active protection of magnesium and its alloys against corrosion.

#### 3.1.4. Evaluation of Inhibitor Release into a Corrosive Medium and Surface Wettability Analysis

**Figure 10:**
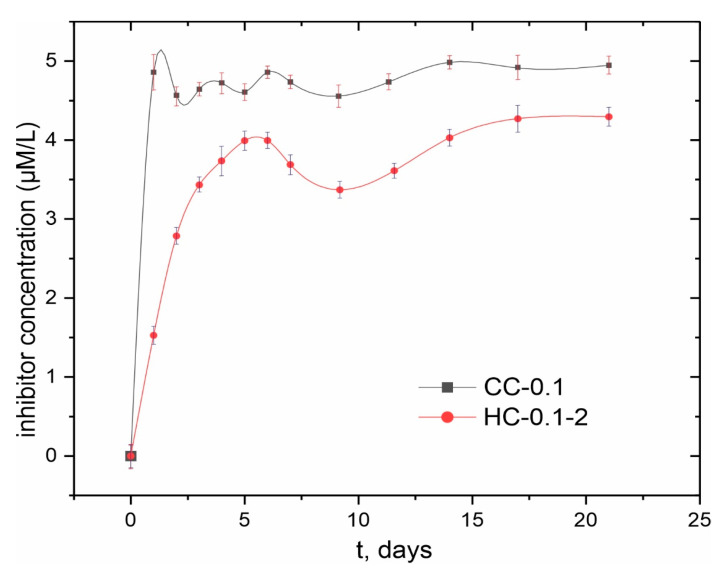
Evolution of the inhibitor release for CC-0.1 and HC-0.1-2 samples exposed to 0.9.wt% NaCl solution for 21 days.

In order to assess the evolution of inhibitor release into a corrosive environment after sealing the coating’s pores with polycaprolactone, the HPLC method was used (Figure 10). An analysis of experimental data indicates a sharp release of benzotriazole into solution on the first day of exposure. With an increase in the immersion time, the concentration of the inhibitor practically does not change. A slight decrease in concentration can be caused by the suppression of the corrosion process during an interaction of benzotriazole released into solution with the surface of magnesium alloy as a result of the partial degradation of the protective layer. It should be noted that the concentration of benzotriazole released during the first day of samples’ exposure did not exceed 5 µM/L, which is about 6 mg/L. Therefore, this dose was not toxic for the human organism (since the median lethal dose LD_50_ = 965 mg/kg [17]).

After treatment of the sample with polycaprolactone, a two-fold decrease in the concentration of the inhibitor released into the solution during the first day of samples’ exposure is observed. The trend of change in inhibitor content in the solution is similar to that observed for the sample with a composite coating. However, compared to samples with CC-0.1 coating, the total amount of benzotriazole released into solution for HC-0.1-2-coated samples remained lower during the experimental period (21 days).

The thickness of the coatings changed after 21 days of exposure to NaCl solution. For CC-0.1 and HC-0.1-2 coatings, the thickness decreased down to 23–26% and 29–35%, respectively (Table S6). In spite of a higher reduction in thickness for HC-0.1-2 as compared to CC-0.1, the hybrid coating is still thicker than the initial polymer-free composite film, indicating that PCL was not completely dissolved from the specimen, according to the data in Table S6. The data obtained indicate a positive effect of the polymer on reducing uncontrolled inhibitor release from the coating composition, which is not associated with the corrosion process. This result confirms the effectiveness of the proposed method for modifying the surface of bioresorbable magnesium alloys. Moreover, it can be taken as a basis when considering dosed drug delivery during the stay of an implant with a hybrid coating in the human body.

Analysis of the surface wettability of the specimens indicates a decrease in the values of the contact angle (CA) after the PEO treatment (CA was 96.9 ± 6.2° and 69.1 ± 1.7° for bare and PEO-coated sample, respectively) due to the formation of a heterogeneous porous structure. Treatment with corrosion inhibitor benzotriazole did not have a significant effect on the surface layer’s wettability (CA was 70.9 ± 2.4°). After the formation of the hybrid coating on the Mg alloy surface, the value of the CA reached up to 85.0 ± 2.0° due to the hydrophobic nature of the polymer. However, the formed surface layer is still hydrophilic. These data are summarized in Figure S5 and Table S7.

### 3.2. Coatings Obtained on AT-Mg

#### 3.2.1. Microstructure Analysis

SEM images of the microstructure of the magnesium sample formed by additive technology (AT-Mg), as well as a cross-section of the PEO coating obtained on its surface, are shown in Figure S6a,b. It should be noted that the microstructure of AT-Mg is represented by magnesium grains with clearly defined zones of the material’s fusion. These areas may be defects that are responsible for the initiation and development of the sample’s corrosion destruction. However, the appearance of the PEO layer formed on the surface of AT-Mg was similar to the one obtained on MA8 wrought Mg alloy.

#### 3.2.2. Electrochemical Properties

**Figure 11:**
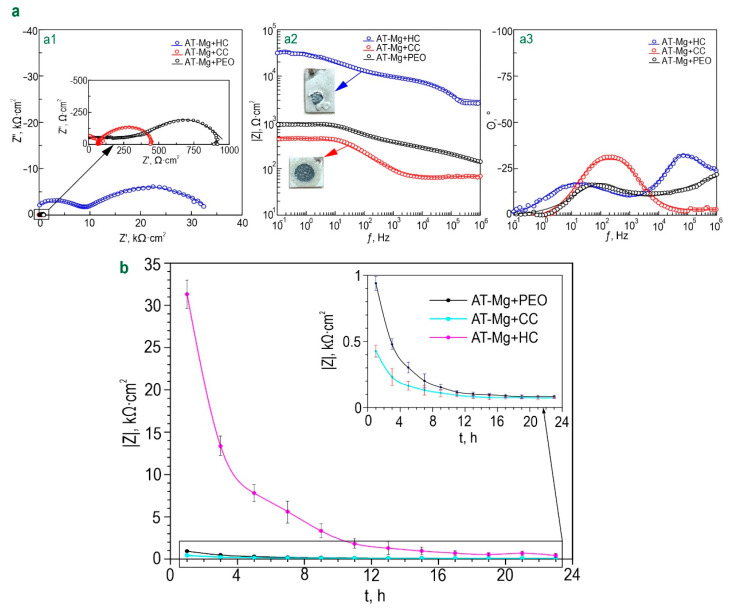
Impedance spectra presented in the form of Nyquist (**a1**) and Bode plots (**a2**,**a3**), obtained after exposure of AT-Mg samples with various types of formed coatings to 0.9% NaCl for 1 h with optical images of samples after long-term (24 h) soaking in sodium chloride solution (**a**) and dynamics of change in the magnitude of impedance modulus, measured at a low frequency (*|Z|_f_*_=0.1Hz_), over exposure time of AT-Mg with different types of coatings to sodium chloride solution (**b**).

The level of corrosion protection of coatings formed on the surface of magnesium obtained by additive technology was established by analyzing the impedance spectra and potentiodynamic polarization curves (Figure 11 and Figure S7). The diagrams of *θ-f* dependence for all types of the studied coatings are characterized by the presence of two time constants (Figure 11a). This determines the fitting of the impedance spectra using an equivalent electrical circuit with two series–parallel *R-CPE* chains (Figure 6e, Table S8). (*R*) and (*CPE*) elements characterize the parameters of the protective layer corresponding to the ones described for coatings formed on the MA8 wrought magnesium alloy. The change in the calculated parameters of the *EEC* for samples with AT-Mg+PEO, as well as benzotriazole-containing coatings for 24 h of exposure to 0.9% NaCl solution, is presented in Table S8. It should be noted that the calculation of the *EEC* parameters for samples with AT-Mg+PEO after 5 h of exposure was not possible due to a significant decrease in the corrosion resistance of the samples and the low quality of the obtained spectra. Analysis of change in the level of corrosion resistance of composite benzotriazole-containing coatings during long-term exposure to 0.9% sodium chloride solution showed a decrease in *|Z|_f_*_=0.1Hz_ as compared to the sample with PEO coating (Figure 11b). This is probably explained by the previously mentioned features of this material. Heterogeneity of the substrate material and the formed oxide layer can provide a decrease in the resistance of the obtained surface layers to corrosion during the process of coating impregnation. The inhibitor concentration used in this study is probably insufficient to provide an increase in corrosion protection for AT-Mg treated by the PEO method. Another possible factor may be the interaction of benzotriazole with a PEO coating matrix, leading to partial dissolution of the protective layer. This effect does not appear for coatings obtained on MA8 magnesium alloy probably due to the different composition and electrochemical and mechanical properties of the substrate materials [34,51]. Nevertheless, in contrast to the destructed AT-Mg+PEO sample, the mechanical integrity of the AT-Mg+CC sample was not broken during exposure to NaCl, which indicates a positive effect of the incorporation of benzotriazole to the composition of the formed coatings.

The results of the assessment of corrosion protection level for samples with benzotriazole-containing coatings by the potentiodynamic polarization method are presented in Table S9 and in Figure S7. The incorporation of benzotriazole into the coating (AT-Mg+CC) contributes to a 2.5-fold decrease in the *I_C_* value (*I_C_* = 4.49 × 10^−5^ A·cm^−2^) in comparison with data for the sample with a PEO layer (*I_C_* = 1.14 × 10^−4^ A·cm^−2^). Some deviations in the results obtained using EIS and PDP can be related to differences in conditions, which were realized when using these electrochemical methods. Polarization during PDP can probably result in more intensive activation of the self-healing properties of the coated sample as compared to EIS measurements, which operated at open circuit potential [52]. Polymer sealing the pores of the oxide layer filled with a corrosion inhibitor contributes to a significant improvement in the resistance of the studied coatings to the occurrence of corrosion processes. A decrease in the value of corrosion current density (*I_C_* = 4.07 × 10^−6^ A·cm^−2^), as well as an increase in the values of impedance modulus measured at a low frequency (*|Z|_f_*_=0.1Hz_ = 31,323 Ω·cm^2^), for a sample with AT-Mg+HC were, respectively, 28 and 33 times that of the AT-Mg+PEO sample (*|Z|_f_*_=0.1Hz_ = 940 Ω·cm^2^, *I_C_* = 1.14·10^−4^ A·cm^–2^ in Table S9). However, the exposure process of the AT-Mg+HC sample is characterized by the variation in *Q* and *n* parameters, which indicates a change in the coating, including morphology, composition, and properties (Table S8). Moreover, if the concentration of the inhibitor introduced into the composition of hybrid coatings is insufficient, sealing the pores of the PEO layer with a polymer component does not always contribute to a significant prolongation of their protective characteristics. However, the optical images shown in Figure 11a confirm a significant decrease in the level of corrosion degradation of AT-Mg+HC samples after 24 h exposure to 0.9% NaCl solution in comparison to other coatings as a result of the best stability of hybrid layers. Therefore, the best protective properties are characterized by the inhibitor- and polymer-containing system obtained on AT-Mg and the MA8 Mg alloy.

The obtained results indicate the successful decrease in the electrochemical activity of both the MA8 magnesium alloy and Mg obtained by additive technology using the targeted formation of polymer-containing self-healing coating. This study contributes to understanding the positive complementary effect of benzotriazole and polycaprolactone in the occurring mechanism of active anticorrosion protection of the Mg-based material. Moreover, it was shown that surface heterogeneity of the PEO coating (including the presence of deep pores) is suitable for filling with modifying agents—biocompatible corrosion inhibitors and polymeric material. Therefore, the porous PEO layer, formed in this work, serves as a good matrix for impregnation with the abovementioned substances. The obtained results indicate that the formed hybrid coatings can provide the level of corrosion protection necessary and sufficient to ensure the mechanical integrity of the surgical implant during bone tissue healing. Thus, such a protective layer can promote the application of magnesium as a biodegradable implant and can enlarge the areas of its practical use in various industries. This study contributes to the further development and design of Mg-based implants.

## 4. Conclusions

To develop a method for modifying the surface of a bioresorbable magnesium alloy (MA8—the Mg-Mn-Ce system—as well as magnesium obtained by additive technology—AT-Mg), hybrid coatings containing an organic biocompatible corrosion inhibitor and a bioresorbable polymer material were formed. Such layers reduce the rate of alloy degradation due to the function of active protection. The following results were obtained:

- Using plasma electrolytic oxidation, a biocompatible ceramic-like coating with a developed surface morphology was obtained. XRD analysis allowed determining periclase (MgO) and forsterite (Mg_2_SiO_4_) in the coating composition;
- The optimal method of surface treatment with benzotriazole (Btr) was selected in order to obtain the most efficient impregnation of PEO layer pores with corrosion inhibitors. The rate of Btr release from the hetero-oxide matrix was reduced by processing the obtained composite coatings with a bioresorbable polymeric material—polycaprolactone. Methods of hybrid coating formation using sequential impregnation of the base PEO layer with Btr at various concentrations and PCL (HC-0.05-2 and HC-0.1-2), as well as one-stage application of Btr and PCL from a solution based on dichloromethane (HC-0.05-1, HC-0.1-1), are presented;
- Using X-ray photoelectron spectroscopy and confocal Raman microspectroscopy, the composition of the formed surface composite inhibitor-containing layers was established. The presence of benzotriazole was confirmed, and its distribution over the coating surface was established at a microlevel;
- The level of corrosion protection of the MA8 magnesium alloy and AT-Mg was determined in 0.9 wt.% NaCl solution. A comparative analysis of the results of electrochemical tests made it possible to establish the best barrier properties for HC-0.1-1 and AT-Mg+HC coatings;
- Analysis of the electrochemical properties of samples with hybrid benzotriazole-containing layers showed stability of their corrosion behavior during 7 days of exposure to Hanks’ solution. Specimens with HC-0.1-1 are also characterized by the smallest volume of released hydrogen and the smallest weight loss among all the studied layers;
- Sealing the pores of the PEO coating with a polymeric material contributes to a significant reduction in inhibitor release into a corrosive medium. The obtained result allows the prolongation of the effect of active anticorrosion protection of the formed hybrid layers. The method of effective hybrid coating formation can provide the required level and duration of the corrosion protection period and controlled degradation of biomedical products from magnesium and its alloys.

## Figures and Tables

**Table 1:** Denominations of coatings formed during the presented study and their descriptions.

Sample’s Designation	Description
PEO	base PEO layer
CC-P	composite coating obtained by treating the PEO layer with 60 g/L polycaprolactone solution in dichloromethane
CC–0.05	composite coating obtained by treating the PEO layer in a 0.05 M benzotriazole aqueous solution
CC–0.1	composite coating obtained by treating the PEO layer in a 0.1 M benzotriazole aqueous solution
HC–0.05-2	hybrid coating obtained in two steps: by treating the PEO layer in a 0.05 M benzotriazole aqueous solution, followed by applying the 60 g/L polycaprolactone in dichloromethane
HC–0.1-2	hybrid coating obtained in two steps: by treating the PEO layer in a 0.1 M benzotriazole aqueous solution, followed by applying the 60 g/L polycaprolactone in dichloromethane
HC–0.05-1	hybrid coating obtained in one step: by treating the PEO layer in the solution of 0.05 M benzotriazole and 60 g/L polycaprolactone in dichloromethane
HC–0.1-1	hybrid coating obtained in one step: by treating the PEO layer in the solution of 0.1 M benzotriazole and 60 g/L polycaprolactone in dichloromethane
AT-Mg+PEO	PEO coating formed on magnesium obtained by additive technology
AT-Mg+CC	composite coating on AT-Mg obtained by impregnating the base PEO layer with 0.1 M benzotriazole aqueous solution
AT-Mg+HC	hybrid coating on AT-Mg obtained in two steps: by impregnating the base PEO layer with 0.1 M benzotriazole aqueous solution, followed by applying the 60 g/L polycaprolactone solution in dichloromethane

**Table 2:** Binding energy (eV) and elemental composition (at.%, in parentheses) of the sample’s surface after treatment of a PEO layer with benzotriazole (CC-0.1).

Element	Chemical State	Studied Surface	
		Before Etching	After Etching (10 min)
Zn (2p)	Zn^2+^	1021.6 (0.2)	1022.4 (0.4)
Na (1s)	Na^+^	1072.2 (0.6)	1072.8 (0.7)
Ce (3d)	Ce^4+^	887.1 (0.2)	887.0 (0.2)
F (1s)	MeF	685.1 (1.8)	686.8 (2.5)
Mn (2p)	Mn^4+^	641.4 (1.1)	642.1 (1.7)
O (1s)	–NO –O–C–	–	534.5 (11.1)
	SiO_2_ O=C–	533.1 (9.2)	533.0 (12.6)
	MgO	531.7 (8.2)	531.5 (5.6)
N (1s)	–N– l	400.4 (10.0)	400.6 (4.1)
	=N-	399.1 (4.7)	399.2 (3.6)
C (1s)	-C(O)O-	–	–
	C–N –C-C(O)O-	286.1 (14.7)	286.4 (12.9)
	C–C, C–H	285.0 (39.1)	285.0 (20.5)
Si (2p)	Si–O	–	105.1 (8.2)
	SiO_2_	103.8 (4.2)	103.4 (4.1)
Mg (2p)	Mg^2+^	–	51.0 (6.0)
	Mg^0^	52.4 (8.7)	49.8 (3.1)

**Table 3:** Electrochemical parameters obtained by analyzing the polarization curves and impedance spectra before and after samples’ exposure to 0.9 wt.% NaCl solution for 24 h.

Coating Type	*β_a_*, mV/decade	–*β_c_*, mV/decade	*I_C_*, A·cm^–2^	*E_C_*, V (Ag/AgCl)	*R_P_*, Ω·cm^2^	*|Z|_f_*_=0.1Hz_, Ω·cm^2^
CC-P before exposure	220.70	218.18	2.40 × 10^−6^	−1.50	1.99 × 10^4^	105,880
CC-P after 24 h of exposure	242.30	96.79	1.43 × 10^−8^	−1.49	2.10 × 10^6^	177,130
CC-0.05 before exposure	263.46	145.81	5.56 × 10^−7^	−1.49	7.35 × 10^4^	61,301
CC-0.05 after 24 h of exposure	932.66	137.04	5.32 × 10^−7^	−1.49	9.93 × 10^4^	51,596
CC-0.1 before exposure	158.29	97.51	1.46 × 10^−7^	−1.44	1.79 × 10^5^	132,050
CC-0.1 after 24 h of exposure	557.88	161.15	1.30 × 10^−7^	−1.38	4.18 × 10^5^	54,112
HC-0.05-2 before exposure	565.54	202.45	3.58 × 10^−7^	−1.45	1.81 × 10^5^	109,830
HC-0.05-2 after 24 h of exposure	479.06	195.30	1.02 × 10^−7^	−1.30	5.90 × 10^5^	46,714
HC-0.1-2 before exposure	484.14	204.88	1.66 × 10^−7^	−1.44	3.77 × 10^5^	403,650
HC-0.1-2 after 24 h of exposure	599.30	199.76	4.97 × 10^−8^	−1.27	1.29 × 10^6^	45,042
HC-0.05-1 before exposure	384.80	181.46	1.00 × 10^−7^	−1.43	5.34 × 10^5^	485,760
HC-0.05-1 after 24 h of exposure	1026.80	218.04	5.27 × 10^−8^	−1.34	1.48 × 10^6^	515,040
HC-0.1-1 before exposure	483.89	274.71	3.43 × 10^−8^	−1.22	2.22 × 10^6^	300,280
HC-0.1-1 after 24 h of exposure	452.04	178.09	3.02 × 10^−8^	−1.35	1.84 × 10^6^	112,470

## Data Availability

Not applicable.

## Supplementary Materials

The following supporting information can be downloaded at: https://www.mdpi.com/article/10.3390/polym15010202/s1, Figure S1: The photographs of samples without coating, with PEO layer, with CC-0.05 and HC-0.05-2 coatings; Figure S2: SEM images of cross-sections and EDX-maps of elements distribution over the thickness of samples with CC-0.05 (a) and CC-0.1 (b) coatings; Figure S3: SEM images of cross-sections and EDX-maps of elements distribution over the thickness of samples with HC–0.05-2 (a) and HC–0.1-2 (b) coatings; Figure S4: SEM images of cross-sections and EDX-maps of elements distribution over the thickness of samples with HC–0.05-1 (a) and HC–0.1-1 (b) coatings; Figure S5: The wettability data: calculated values of the contact angle and images of the drops on the surface of the bare Mg alloy sample, specimen with PEO layer, with CC-0.05 and HC-0.05-2 coatings; Figure S6: SEM images of the microstructure of a magnesium sample obtained by additive technology (a) and a PEO coating formed on AT-Mg (b); Figure S7: Polarization curves obtained for AT-Mg samples with different types of coatings, before (a) and after (b) 24 h exposure to 0.9% sodium chloride solution; Table S1: Calculated parameters of equivalent electrical circuits (EEC) elements, obtained by fitting the impedance spectra of samples with PEO and CC-P during the exposure to 0.9% NaCl solution; Table S2: Calculated parameters of equivalent electrical circuits (EEC) elements, obtained by fitting the impedance spectra of a sample with different types of coatings during the exposure to 0.9% NaCl solution; Table S3: Calculated parameters of polarization curves obtained for samples with HC-0.1-2 before and after immersion in HBSS; Table S4: Calculated parameters of equivalent electrical circuit (EEC) elements, obtained by fitting the impedance spectra of a sample with HC-0.1-2 coating during the exposure to HBSS for 7 days; Table S5: The results of gravimetric tests of the studied coated samples in Hanks solution (HBSS) for 7 days; Table S6: Thicknesses of different coatings calculated before and after the exposure to 0.9% NaCl for 21 days; Table S7: Wettability data for samples with different coatings; Table S8: Calculated parameters of equivalent electrical circuit (EEC) elements, obtained by fitting the impedance spectra of AT-Mg samples with BTR-containing coatings during the exposure to 0.9% NaCl; Table S9: Calculated parameters of polarization curves obtained for AT-Mg samples with Btr-containing coatings before and after immersion in 0.9% NaCl for 24 h.
