# Supplementary material for: New Polycaprolactone-Containing Self-Healing Coating Design for Enhance Corrosion Resistance of the Magnesium and Its Alloys
Source: Polymers (Basel). 2022 Dec 31;15(1):202. doi: 10.3390/polym15010202 (PMC9824560; doi:10.3390/polym15010202)
Supplement: Supplementary file 1 [file polymers-15-00202-s001.zip › polymers-2073581-supplementary.pdf]

polymers

MDPI

Supplementary Materials

New Polycaprolactone-Containing Self-Healing Coating Design for Enhance Corrosion Resistance of the Magnesium and Its Alloys

Andrey S. Gnedenkov *, Sergey L. Sinebryukhov, Valeriia S. Filonina, Alexander Yu. Ustinov, Sviatoslav V. Sukhoverkhov and Sergey V. Gnedenkov

Institute of Chemistry, Far Eastern Branch of the Russian Academy of Sciences, 690022 Vladivostok, Russia
* Correspondence: asg17@mail.com; Tel.: +8-(423)-2215284; Fax: +8-(423)-2312590

SUPPLEMENTARY MATERIALS (FIGURES)

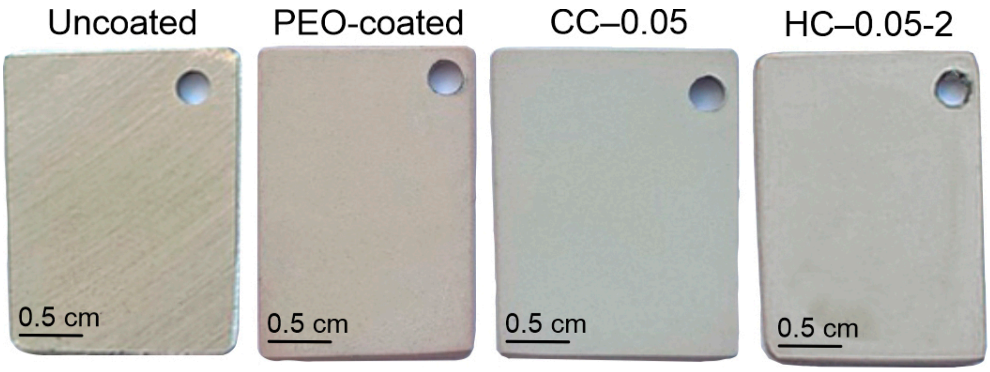

Figure S1. The photographs of samples without coating, with PEO layer, with CC-0.05 and HC-0.05-2 coatings.

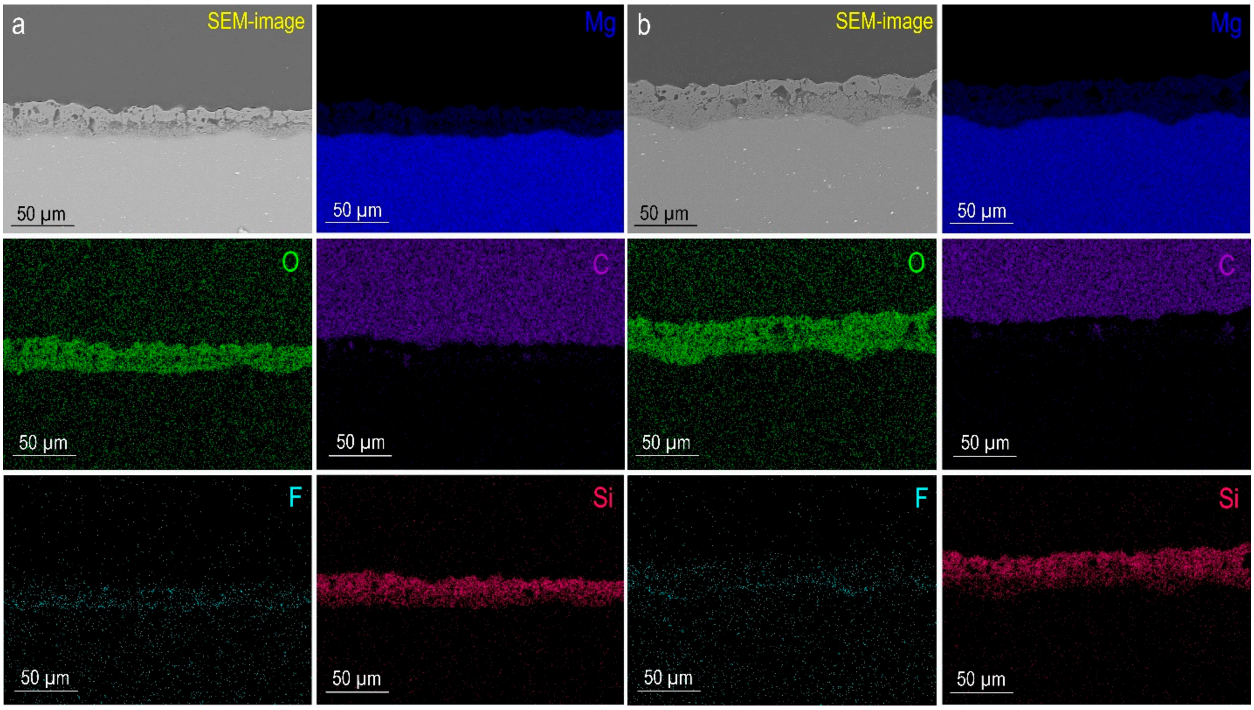

Figure S2. SEM images of cross-sections and EDX-maps of elements distribution over the thickness of samples with CC-0.05 (a) and CC-0.1 (b) coatings.

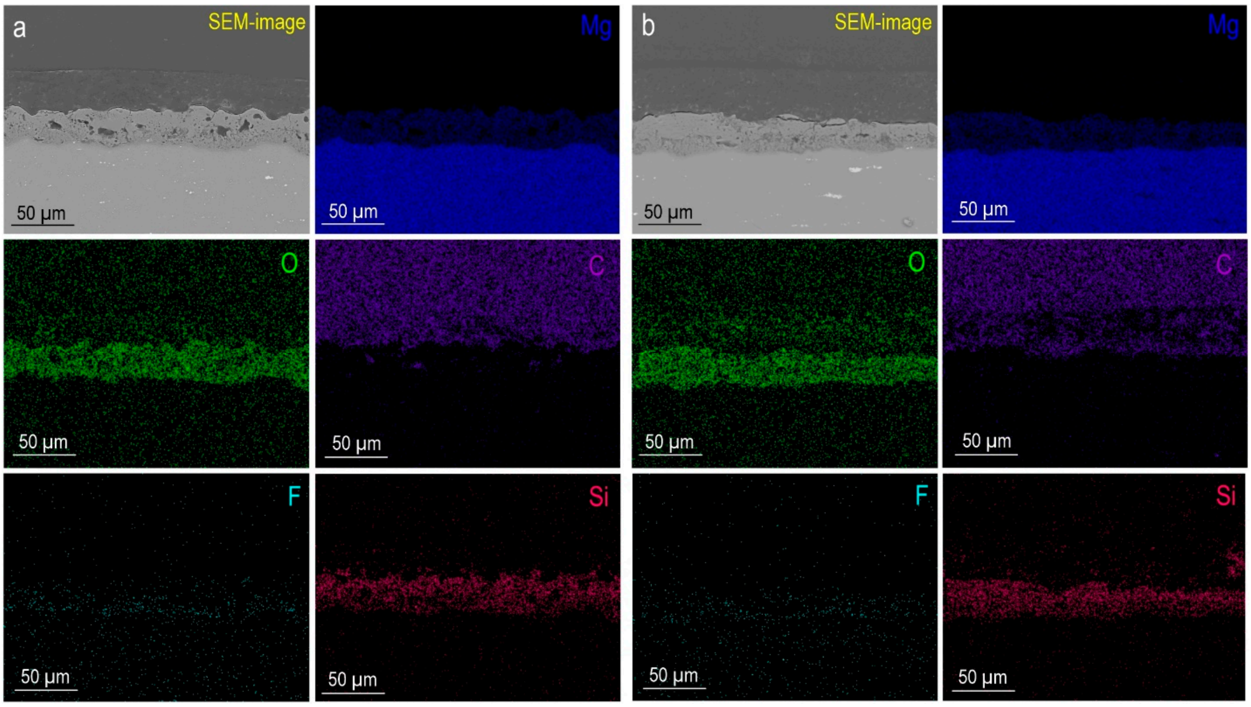

Figure S3. SEM images of cross-sections and EDX-maps of elements distribution over the thickness of samples with HC–0.05-2 (a) and HC–0.1-2 (b) coatings.

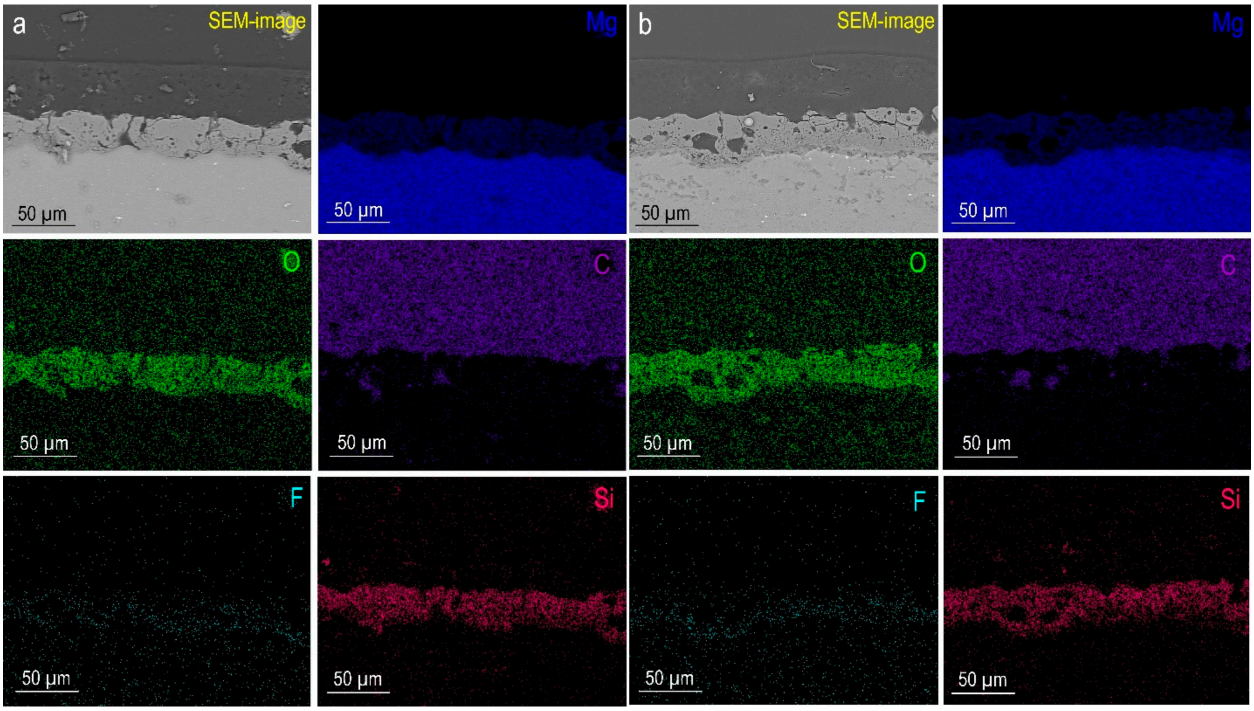

Figure S4. SEM images of cross-sections and EDX-maps of elements distribution over the thickness of samples with HC–0.05-1 (a) and HC–0.1-1 (b) coatings.

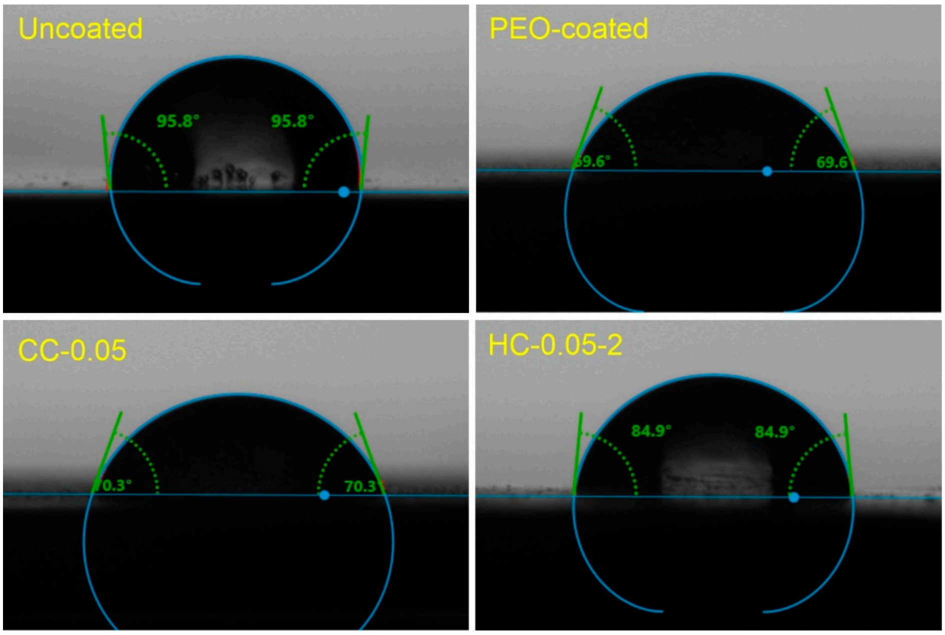

Figure S5. The wettability data: calculated values of the contact angle and images of the drops on the surface of the bare Mg alloy sample, specimen with PEO layer, with CC-0.05 and HC-0.05-2 coatings.

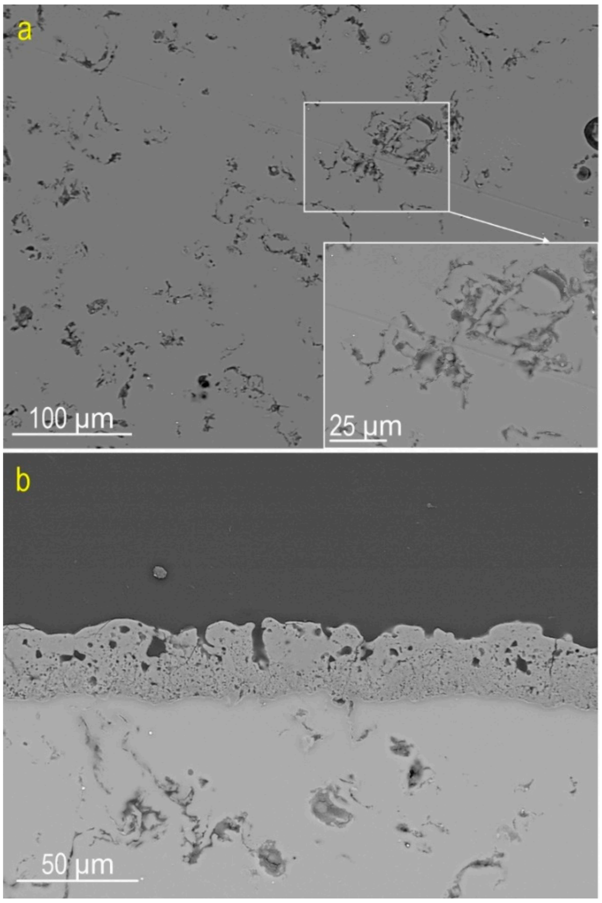

Figure S6. SEM images of the microstructure of a magnesium sample obtained by additive technology (a) and a PEO coating formed on AT-Mg (b).

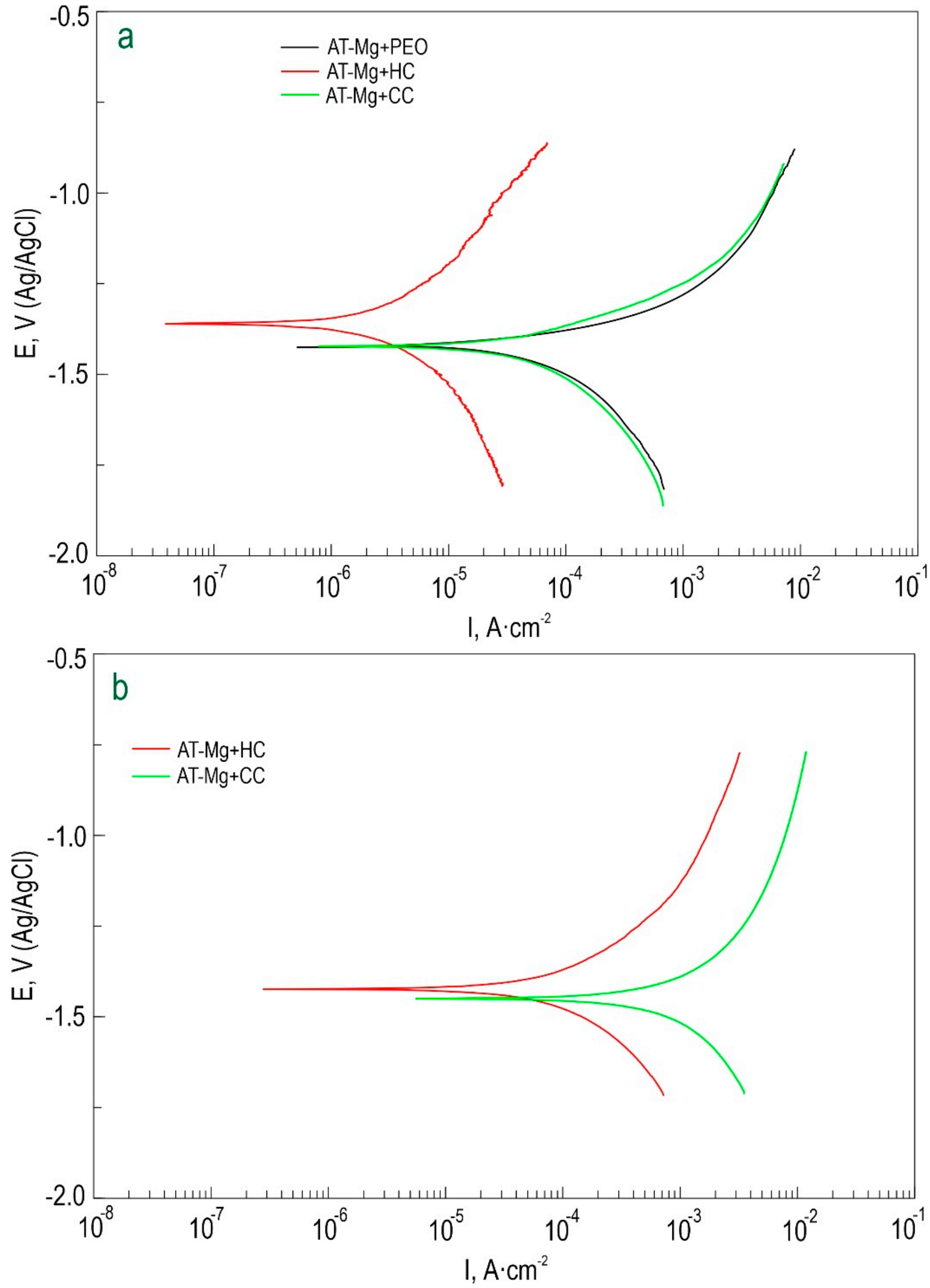

Figure S7. Polarization curves obtained for AT-Mg samples with different types of coatings, before (a) and after (b) 24 h exposure to 0.9% sodium chloride solution.

SUPPLEMENTARY MATERIALS (TABLES)

Table S1. Calculated parameters of equivalent electrical circuits (EEC) elements, obtained by fitting the impedance spectra of samples with PEO and CC-P during the exposure to 0.9% NaCl solution.

Exposure time, h	CPE_1		R_1, Ω·cm²	CPE_2		R_2, Ω·cm²
	Q_1, S·cm⁻²·sⁿ	n_1		Q_2, S·cm⁻²·sⁿ	n_2	
PEO						
1	8.41×10^{-7}	0.61	2,897	6.46×10^{-7}	0.91	77,559
3	6.75×10^{-7}	0.62	2,362	1.31×10^{-6}	0.81	73,670
5	7.78×10^{-7}	0.61	2,243	1.31×10^{-6}	0.82	86,070
7	4.14×10^{-7}	0.65	1,554	1.53×10^{-6}	0.80	67,398
9	4.20×10^{-7}	0.65	1,589	1.23×10^{-6}	0.84	82,627
11	5.78×10^{-7}	0.63	1,670	1.21×10^{-6}	0.83	65,201
13	4.55×10^{-7}	0.64	1,481	2.17×10^{-6}	0.73	49,428
15	7.32×10^{-7}	0.61	1,628	1.12×10^{-6}	0.86	63,934
17	4.70×10^{-7}	0.64	1,266	1.77×10^{-6}	0.78	50,641
19	9.11×10^{-7}	0.58	1,554	1.09×10^{-6}	0.85	69,427
21	1.36×10^{-6}	0.57	1,701	9.33×10^{-7}	0.86	78,171
23	2.23×10^{-6}	0.54	1,869	4.53×10^{-7}	0.95	60,522
CC-P						
1	4.41×10^{-9}	0.84	5,229	7.68×10^{-7}	0.78	112,710
3	4.31×10^{-9}	0.84	7,507	8.78×10^{-7}	0.77	91,727
5	4.47×10^{-9}	0.84	7,558	9.33×10^{-7}	0.78	73,998
7	5.09×10^{-9}	0.83	7,652	1.00×10^{-6}	0.74	109,420
9	4.87×10^{-9}	0.83	8,574	1.10×10^{-6}	0.71	122,800
11	5.25×10^{-9}	0.82	9,178	9.34×10^{-7}	0.75	112,040
13	4.84×10^{-9}	0.83	9,449	1.18×10^{-6}	0.69	120,070
15	5.68×10^{-9}	0.81	9,791	1.00×10^{-6}	0.74	108,650
17	5.45×10^{-9}	0.82	9,961	1.38×10^{-6}	0.67	115,060
19	6.14×10^{-9}	0.81	10,241	1.37×10^{-6}	0.65	138,260
21	5.51×10^{-9}	0.81	10,846	1.22×10^{-6}	0.68	133,120
23	6.37×10^{-9}	0.80	11,011	1.39×10^{-6}	0.63	160,000

Table S2. Calculated parameters of equivalent electrical circuits (EEC) elements, obtained by fitting the impedance spectra of a sample with different types of coatings during the exposure to 0.9% NaCl solution.

Exposure time, h	CPE_1		R_1, Ω·cm²	CPE_2		R_2, Ω·cm²
	Q_1, S·cm⁻²·sⁿ	n_1		Q_2, S·cm⁻²·sⁿ	n_2	
CC-0.05						
1	2.30×10^{-6}	0.90	59,935			
3	2.16×10^{-6}	0.92	73,753			
5	2.15×10^{-6}	0.92	89,834			
7	2.17×10^{-6}	0.92	92,959			
9	2.22×10^{-6}	0.92	82,272			
11	2.26×10^{-6}	0.92	82,164			
13	2.30×10^{-6}	0.92	76,109			
15	2.34×10^{-6}	0.92	72,135			
17	2.37×10^{-6}	0.92	68,145			
19	2.39×10^{-6}	0.92	64,875			
21	2.42×10^{-6}	0.92	62,891			
23	2.60×10^{-6}	0.93	46,528			
CC-0.1						
1	2.23×10^{-6}	0.85	142,730			
3	2.12×10^{-6}	0.86	194,210			
5	2.13×10^{-6}	0.87	220,160			
7	2.15×10^{-6}	0.87	351,200			
9	2.26×10^{-6}	0.86	160,080			
11	2.56×10^{-6}	0.85	96,466			
13	2.42×10^{-6}	0.86	51,996			
15	2.49×10^{-6}	0.86	96,109			
17	2.43×10^{-6}	0.86	148,080			
19	2.61×10^{-6}	0.86	74,918			
21	2.48×10^{-6}	0.86	65,877			
23	2.63×10^{-6}	0.86	60,707			
HC-0.05-2						
1	2.53×10^{-9}	0.95	964	2.81×10^{-6}	0.78	119,850
3	2.64×10^{-9}	0.95	966	2.85×10^{-6}	0.78	233,810
5	1.77×10^{-9}	0.97	1,044	2.83×10^{-6}	0.78	305,820

7	1.84×10^{-9}	0.97	1,101	2.81×10^{-6}	0.79	361,950
9	1.79×10^{-9}	0.97	1,103	3.10×10^{-6}	0.77	199,280
11	1.58×10^{-9}	0.98	1,018	4.14×10^{-6}	0.72	94,020
13	2.03×10^{-9}	0.97	1,004	3.55×10^{-6}	0.75	69,409
15	2.45×10^{-9}	0.94	1,005	3.37×10^{-6}	0.77	40,194
17	3.54×10^{-9}	0.93	1,062	3.26×10^{-6}	0.77	84,749
19	2.59×10^{-9}	0.95	943	3.37×10^{-6}	0.76	75,167
21	2.00×10^{-9}	0.97	918	3.72×10^{-6}	0.74	56,554
23	1.82×10^{-9}	0.98	834	4.32×10^{-6}	0.72	60,332
			HC-0.1-2			
1	4.44×10^{-8}	0.72	3,198	1.61×10^{-6}	0.76	534,870
3	6.17×10^{-8}	0.71	2,831	2.07×10^{-6}	0.72	320,580
5	8.58×10^{-8}	0.68	3,143	1.71×10^{-6}	0.78	332,250
7	7.40×10^{-8}	0.69	3,181	1.69×10^{-6}	0.79	258,670
9	9.19×10^{-8}	0.68	3,109	1.84×10^{-6}	0.78	194,880
11	1.34×10^{-7}	0.65	3,193	1.75×10^{-6}	0.81	215,100
13	1.29×10^{-7}	0.65	3,042	1.63×10^{-6}	0.82	188,100
15	1.94×10^{-7}	0.63	2,988	1.79×10^{-6}	0.82	111,690
17	1.75×10^{-7}	0.64	2,597	2.01×10^{-6}	0.81	81,176
19	1.49×10^{-7}	0.66	2,164	2.22×10^{-6}	0.78	70,944
21	1.40×10^{-7}	0.67	1,900	2.19×10^{-6}	0.78	69,936
23	1.81×10^{-7}	0.65	1,886	2.10×10^{-6}	0.80	45,485
			HC-0.05-1			
1	3.54×10^{-8}	0.75	4,549	1.39×10^{-6}	0.71	589,900
3	1.39×10^{-7}	0.63	8,530	1.24×10^{-6}	0.73	506,840
5	1.15×10^{-7}	0.65	10,129	1.18×10^{-6}	0.74	453,430
7	1.15×10^{-7}	0.65	11,347	1.09×10^{-6}	0.76	437,310
9	7.35×10^{-8}	0.68	10,128	1.52×10^{-6}	0.67	423,120
11	9.09×10^{-8}	0.66	11,180	1.31×10^{-6}	0.71	335,400
13	7.32×10^{-8}	0.68	10,648	1.54×10^{-6}	0.67	349,390
15	6.15×10^{-8}	0.69	8,535	1.69×10^{-6}	0.67	290,980
17	1.09×10^{-7}	0.65	7,970	1.34×10^{-6}	0.70	231,360
19	1.55×10^{-7}	0.63	7,640	1.20×10^{-6}	0.71	208,110
21	8.75×10^{-8}	0.67	6,916	1.79×10^{-6}	0.65	198,100
23	1.18×10^{-7}	0.65	4,720	1.69×10^{-6}	0.67	129,170

HC-0.1-1						
1	$4.37×10^{-7}$	0.58	4,559	$8.28×10^{-7}$	0.75	362,120
3	$2.00×10^{-7}$	0.61	7,651	$9.60×10^{-7}$	0.73	640,440
5	$1.15×10^{-7}$	0.64	10,454	$9.18×10^{-7}$	0.74	616,790
7	$7.71×10^{-8}$	0.66	11,719	$9.69×10^{-7}$	0.73	584,540
9	$5.31×10^{-8}$	0.69	11,118	$1.30×10^{-6}$	0.66	492,160
11	$4.47×10^{-8}$	0.70	11,210	$1.34×10^{-6}$	0.66	390,790
13	$3.27×10^{-8}$	0.72	10,636	$1.56×10^{-6}$	0.64	300,100
15	$3.69×10^{-8}$	0.71	10,563	$1.22×10^{-6}$	0.71	416,160
17	$1.68×10^{-8}$	0.77	12,275	$1.96×10^{-6}$	0.62	688,130
19	$2.20×10^{-8}$	0.75	10,277	$1.52×10^{-6}$	0.68	579,040
21	$1.53×10^{-8}$	0.77	9,016	$1.64×10^{-6}$	0.67	473,270
23	$3.02×10^{-8}$	0.89	8,897	$2.71×10^{-6}$	0.57	187,790

Table S3. Calculated parameters of polarization curves obtained for samples with HC-0.1-2 before and after immersion in HBSS.

**Coating type**	β_a**, mV/decade**	$-\beta_c$**, mV/decade**	I_C**, A·cm^{-2}**	E_C**, V (Ag/AgCl)**	R_P**, Ω·cm^2**	$\lvert Z\rvert_{f=0.1\ Hz}$**, Ω·cm^2**
HC–0.1-2 before immersion	664.50	230.31	$8.90·10^{-7}$	-1.61	$8.36·10^{4}$	$7.82·10^{5}$
HC–0.1-2 after immersion	404.16	187.48	$3.12·10^{-8}$	-1.36	$1.78·10^{6}$	$1.52·10^{5}$

Table S4. Calculated parameters of equivalent electrical circuit (EEC) elements, obtained by fitting the impedance spectra of a sample with HC-0.1-2 coating during the exposure to HBSS for 7 days.

**Immersion time, h**	***CPE*$_1$**		R_1**, Ω·cm^2**	***CPE*$_2$**		R_2**, Ω·cm^2**
	Q_1**, S·cm^{-2}·s^n**	n_1		Q_2**, S·cm^{-2}·s^n**	n_2	
1	$1.59×10^{-8}$	0.71	29,412	$6.28×10^{-6}$	0.52	70,115
3	$2.51×10^{-9}$	0.87	13,005	$4.05×10^{-6}$	0.61	105,320
11	$8.27×10^{-9}$	0.86	8,337	$4.11×10^{-6}$	0.71	335,460
23	$3.07×10^{-8}$	0.75	9,155	$3.97×10^{-6}$	0.77	370,850
47	$2.47×10^{-7}$	0.58	13,200	$2.83×10^{-6}$	0.91	456,370

71	2.32×10^{-7}	0.58	12,700	3.42×10^{-6}	0.81	402,100
119	2.67×10^{-7}	0.58	12,937	2.79×10^{-6}	0.91	422,900
167	2.25×10^{-8}	0.77	9,560	4.50×10^{-6}	0.75	244,630

Table S5. The results of gravimetric tests of the studied coated samples in Hanks solution (HBSS) for 7 days.

PEO				
Sample (№)	1	2	3	4
Weight before immersion, g	0.5949	0.6908	0.5903	0.6393
Weight after immersion, g	0.5929	0.6887	0.5885	0.6370
Difference, g	0.0020	0.0021	0.0018	0.0023
Mass loss, $mg\cdot cm^{-2}\cdot day^{-1}$	0.0405	0.0426	0.0365	0.0466
**CC-P**				
Sample (№)	1	2	3	4
Weight before immersion, g	0.5491	0.6727	0.6919	0.6998
Weight after immersion, g	0.5502	0.6736	0.6929	0.7007
Mass increase, g	0.0011	0.0009	0.0010	0.0009
**CC-0.05**				
Sample (№)	1	2	3	4
Weight before immersion, g	0.5974	0.6201	0.6438	0.6506
Weight after immersion, g	0.5959	0.6191	0.6422	0.6491
Difference, g	0.0015	0.0010	0.0016	0.0015
Mass loss, $mg\cdot cm^{-2}\cdot day^{-1}$	0.0304	0.0203	0.0324	0.0304
**CC-0.1**				
Sample (№)	1	2	3	4
Weight before immersion, g	0.6726	0.6673	0.6194	0.6182
Weight after immersion, g	0.6714	0.6662	0.6178	0.6162
Difference, g	0.0012	0.0011	0.0016	0.0020
Mass loss, $mg\cdot cm^{-2}\cdot day^{-1}$	0.0243	0.0223	0.0324	0.0405
**HC-0.05-2**				
Sample (№)	1	2	3	4
Weight before immersion, g	0.6543	0.6165	0.6658	0.6230

Weight after immersion, g	0.6548	0.6168	0.6662	0.6232
Mass increase, g	0.0005	0.0003	0.0004	0.0002
HC-0.1-2				
Sample (№)	1	2	3	4
Weight before immersion, g	0.7327	0.6980	0.6938	0.7145
Weight after immersion, g	0.7328	0.6981	0.6940	0.7148
Mass increase, g	0.0001	0.0001	0.0002	0.0003
HC-0.05-1				
Sample (№)	1	2	3	4
Weight before immersion, g	0.6890	0.6652	0.7466	0.6992
Weight after immersion, g	0.6863	0.6631	0.7441	0.6968
Difference, g	0.0027	0.0021	0.0025	0.0024
Mass loss, mg·cm^{-2}·day^{-1}	0.0547	0.0426	0.0507	0.0486
HC-0.1-1				
Sample (№)	1	2	3	4
Weight before immersion, g	0.6122	0.6207	0.6612	0.6721
Weight after immersion, g	0.6108	0.6188	0.6598	0.6709
Difference, g	0.0014	0.0019	0.0014	0.0012
Mass loss, mg·cm^{-2}·day^{-1}	0.0284	0.0385	0.0284	0.0243

Table S6. Thicknesses of different coatings calculated before and after the exposure to 0.9% NaCl for 21 days.

Coating type		Thickness, μm
CC-0.1 before exposure		38.8±2.3
CC-0.1 after exposure	Sample 1	28.6±2.1
	Sample 2	29.1±3.2
	Sample 3	30.0±2.7
	Sample 4	29.3±3.1
HC-0.1-2 before exposure		69.9±4.7
HC-0.1-2 after exposure	Sample 1	49.6±4.7
	Sample 2	45.1±4.7
	Sample 3	46.3±5.5
	Sample 4	45.4±2.5

Table S7. Wettability data for samples with different coatings.

Coating type	Contact angle, °
Uncoated	96.9±6.2
PEO-coated	69.1±1.7
CC-0.05	70.9±2.4
HC-0.05-2	85.0±2.0

Table S8. Calculated parameters of equivalent electrical circuit (*EEC*) elements, obtained by fitting the impedance spectra of AT-Mg samples with BTR-containing coatings during the exposure to 0.9% NaCl.

Immersion time, h	CPE_1		R_1, Ω·cm²	CPE_2		R_2, Ω·cm²
	Q_1, S·cm^{-2}·s^n	n_1		Q_2, S·cm^{-2}·s^n	n_2	
AT-Mg+PEO						
1	2.50×10^{-6}	0.49	252	9.58×10^{-5}	0.47	795
3	1.20×10^{-6}	0.52	179	9.47×10^{-5}	0.69	290
5	1.08×10^{-6}	0.50	129	9.47×10^{-4}	0.82	141
AT-Mg+CC						
1	7.98×10^{-9}	0.95	68	4.06×10^{-5}	0.72	410
3	7.63×10^{-9}	0.95	64	1.72×10^{-4}	0.58	204
5	7.19×10^{-9}	0.96	58	4.52×10^{-4}	0.49	131
AT-Mg+HC						
1	5.95×10^{-8}	0.62	9,588	7.45×10^{-6}	0.48	27,455
3	2.69×10^{-8}	0.70	4,750	1.24×10^{-5}	0.49	9,722
5	3.22×10^{-8}	0.69	3,256	2.44×10^{-5}	0.49	5,464
7	2.89×10^{-9}	0.88	2,194	3.38×10^{-5}	0.49	3,603
9	6.72×10^{-10}	0.99	1,621	4.72×10^{-5}	0.53	1,906
11	8.25×10^{-10}	0.99	1,007	2.86×10^{-4}	0.36	1,175
13	9.41×10^{-10}	0.99	780	8.71×10^{-4}	0.39	844
15	1.10×10^{-9}	0.99	557	6.95×10^{-4}	0.37	620
17	1.19×10^{-9}	0.99	397	8.03×10^{-4}	0.33	456
19	1.28×10^{-9}	0.99	350	2.01×10^{-3}	0.29	435
21	1.33×10^{-9}	0.99	304	2.07×10^{-3}	0.29	383
23	1.41×10^{-9}	0.99	258	2.37×10^{-3}	0.28	299

Table S9. Calculated parameters of polarization curves obtained for AT-Mg samples with Btr-containing coatings before and after immersion in 0.9 % NaCl for 24 h.

**Coating type**	β_a, **mV/decade**	$-\beta_c$, **mV/decade**	I_C, **A·cm^{-2}**	E_C, **V (Ag/AgCl)**	R_P, **Ω·cm^2**	$\lvert Z \rvert_{f=0.1\ Hz}$, **Ω·cm^2**
Before immersion						
AT-Mg+PEO	275.45	556.01	1.14×10^{-4}	-1.45	7.02×10^{2}	940
AT-Mg+CC	124.31	236.40	4.49×10^{-5}	-1.42	7.88×10^{2}	426
AT-Mg+HC	399.02	433.52	4.07×10^{-6}	-1.36	2.22×10^{4}	31,323
After immersion						
AT-Mg+PEO	–	–	–	–	–	82
AT-Mg+CC	360.58	430.33	9.59×10^{-4}	-1.45	8.89×10^{1}	74
AT-Mg+HC	175.35	183.52	5.93×10^{-5}	-1.42	6.57×10^{2}	418
